# Oil-Spill Triggered Shift in Indigenous Microbial Structure and Functional Dynamics in Different Marine Environmental Matrices

**DOI:** 10.1038/s41598-018-37903-x

**Published:** 2019-02-04

**Authors:** C. S. Neethu, C. Saravanakumar, R. Purvaja, R. S. Robin, R. Ramesh

**Affiliations:** National Centre for Sustainable Coastal Management (NCSCM), Ministry of Environment, Forest and Climate Change (MoEFCC), Chennai, 600025 India

## Abstract

Microbial degradation has long been recognized as the key rescue mechanism in shaping the oil polluted marine environments and the role of indigenous populations or their functional genomics have never been explored from Indian marine environments, post an oil spill event. In the current study, high throughput metagenomic analysis, PLFA profiling and mass spectrophotometric analysis was performed in combination with metabolomics to capture signature variations among the microbial communities in sediment, water and laboratory enrichments. Contrary to the previous reports, the bloom of Pseudomonadales (specifically genus *Acinetobacter*) in oiled sediment and Methylococcales in oiled water outnumbered the relative abundance of *Alcanivorax* in response to hydrocarbon contamination. Overall enhancement of xenobiotic degradation was suggested by metabolomic analysis in sediment and water post the spill event and varying quantitative assemblage of enzymes were found to be involved in hydrocarbon utilization. Laboratory enrichments revealed the competitive advantage of sediment communities over the water communities although unique taxa belonging to the later were also found to be enriched under *in vitro* conditions. Simultaneous analysis of sediment and water in the study provided explicit evidences on existence of differential microbial community dynamics, offering insight into possibilities of formulating nature identical solutions for hydrocarbon pollution.

## Introduction

Contamination of marine environment with crude oil spillage is estimated to be over a million tons per year globally, causing sustained and severe impacts on the ecosystems and food webs^1–3^. Mitigation of spread of spilled oil depends on factors including composition of oil, temperature, oxygen availability, mechanical removal and most significantly natural or artificial enrichment of biodegrading microbial population^4–6^. Metabolic plasticity allows microbial communities to scavenge on a wide variety of complex hydrocarbon substrates leading to the effective removal of spilled oil from the natural environment^7–9^. Spill accidents close to the shore have immediate impacts on different environmental matrices including sediment and water ecosystems and have rarely been studied together.

The coastline of India covers approximately 7500 km and had been impacted by three reported spill accidents in marine ecosystems between 2010 and 2017 predominantly along the west coast of India. The study reported here, is the first major oil spill incident from the east coast of India. Incidentally, microbial community dynamics or their functional interpretations in these oil impacted marine habitats are relatively under studied. On January 28, 2017, collision of ships Don Kanchivaram with BW Maple at the Chennai coast (80°21′46″E, 13°13′41″N) of India released about 196 metric tons of heavy oil into the Bay of Bengal^10^. An estimate of about 180.89 km^2^ was the areal spread of the heavy oil as on 31 January 2017 based on satellite image processing^10^. The southwest ward movement of current, transported the spilled oil towards south and deposited a fraction of it as tarballs ashore. Even though majority of the spilled oil was removed physically, a significant portion of remnant oil in the containment booms in the bay and tarballs that were washed ashore posed challenges in complete elimination of these pollutants from the marine environment.

The ultimate fate of these pollutants in the marine environment is determined by differential potential of microbial communities in individual niches or their synergetic effects^11–14^. Although hydrocarbon degrading populations are ubiquitous in distribution in marine environment, they exist as a minor fraction of the pre spill community^4^. Reported studies indicate the bio-geographical distribution of these hydrocarbonoclastic bacteria, especially predominance of genera *Alcanivorax*, *Thalassolituus*, *Cycloclasticus*, *Pseudomonas*, *Thalasomonas*, *Sulfitobacter*, *Oleibacter* and *Oleispira* from diverse oil polluted marine environment^7,11,12,15^. Oceanic blooms of *Cycloclasticus*, *Oceanospirillales*, *Pseudoalteromonas*, *Sulfitobacter*, *Thalassospira and Roseobacter* are known to be associated with intrinsic hydrocarbon removal from seawater^16–19^. In sediments, microbial succession that develop into hydrocarbon degrading consortia, have been paralleled with enhanced mixing of nutrients and oxygen^20,21^ involving autochthonous degraders comprising of Desulfobacteraceae, Methylococcaceae, Methylophilaceae, Desulfuromonadaceae, *Hyphomonas*, *Pseudomonas*, *Marinobacter*, *Methylophaga*, *Sulfurimonas* and *Helicobacter*^20,22–24^. Despite the significant role of predominant genera, variable combinations of bacterial and fungal networking with complementary catabolic competences could be involved in complete biodegradation of these pollutants. Phylogenetic affiliations belonging to a few fungal orders (Agaricales, Saccharomycetales and Hypocreales) and species such as *Trichoderma* spp., *Penicillium* spp., *Phanerochaete chrysosporum*, *Pleurotus ostreatus*, *Lactiporus sulphurus* and *Flammulina velutipes* have been reported to degrade hydrocarbons effectively^25–28^. In the current study, stochastic responses of heterogeneity in fungal and bacterial communities in response to influx of hydrocarbon have been investigated for the first time from tropical marine environment.

Reconstruction of broader networks between functional microbes that directly utilize hydrocarbon and those that depend on secondary metabolites or intermediates would aid in highlighting the complexity of interactions between microbial communities^29–31^. Even though constrains of predictive analysis exist with PICRUSt and KEGG annotations, an attempt was made with the information generated from PICRUSt, KEGG orthologs and gene expression analysis to draw an understanding on functionalities of microbial communities. To our knowledge, previously identified diversity dynamics and functional characterization on microbial responses have always been from single environmental matrix and this study is perhaps the first to address such responses on different environmental matrices including laboratory enrichments. We attempt to gain new insights in to these aspects by a comprehensive analysis of the three systems (a) oil contaminated seawater- sediment (b) non-oil contaminated seawater- sediment and (c) *in vitro* enrichments to address the following questions- (1) How different is the percentage composition of bacterial and fungal communities in sediment and seawater in response to oil contamination when analyzed simultaneously from the same site to that of the previously reported independent analysis? (2) Whether similar trends exist in artificial and natural enrichment in taxonomic variability and functional preferences? (3) What percentage of indigenous microbial population that degrade hydrocarbon could possibly be obtained in pure culture to form efficient consortia? and (4) What difference exists in the hydrocarbon degradation potential of various microbial enrichments that could be used for future applications?

## Results

### Physicochemical analysis

**Figure 1:**
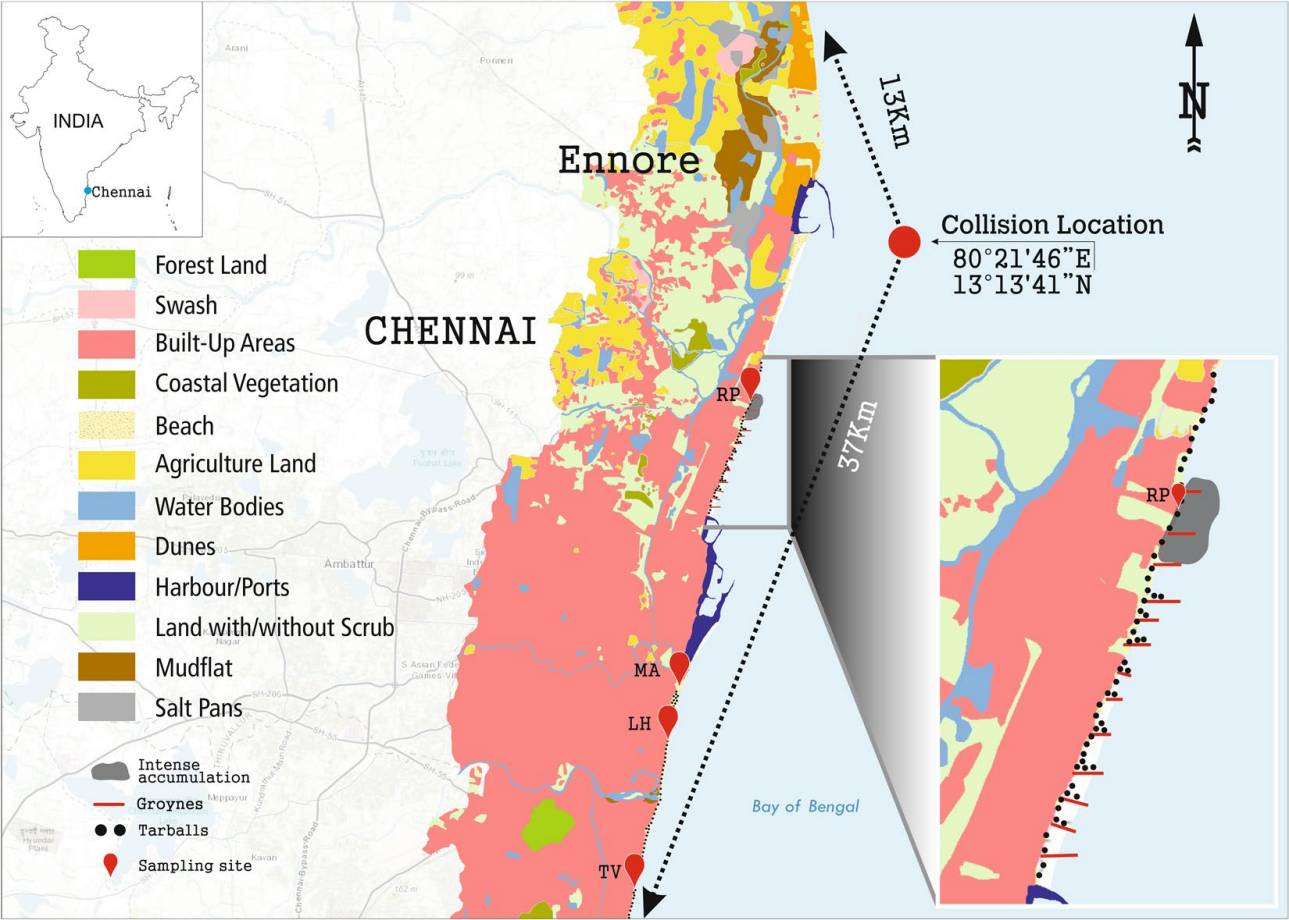
Location map showing ship collision site and sampling sites based on remote sensing. The samples were collected at Royapuram (RP), Marina (MA), Light House (LH) and Thiruvanmiyur (TV). Enlarged view represents intense accumulation of spilled oil contained in boom (at RP) and spread of tarballs towards south of Chennai coast. Arc GIS (version 10.5.1, http://desktop.arcgis.com/en/arcmap/) was used for creating the map.

Even **s**ubsequent to the spill, no substantial change was observed in ambient seawater temperature, pH, or salinity. There were minor variations in dissolved oxygen concentration and oxygen saturation between the oiled and non-oiled samples (Supplementary Table S1). Elemental (CHNS) analysis of oiled and non-oiled sediments revealed increase of carbon, hydrogen and sulphur in the oiled samples after the spill event, as expected (Supplementary Table S2). No physical or chemical factors other than hydrocarbons were substantially altered. Wind speed (1.9 m/s) indicated the movement and direction of spill towards southwest (Fig. 1).

### Growth enrichment and hydrocarbon content analysis

**Figure 2:**
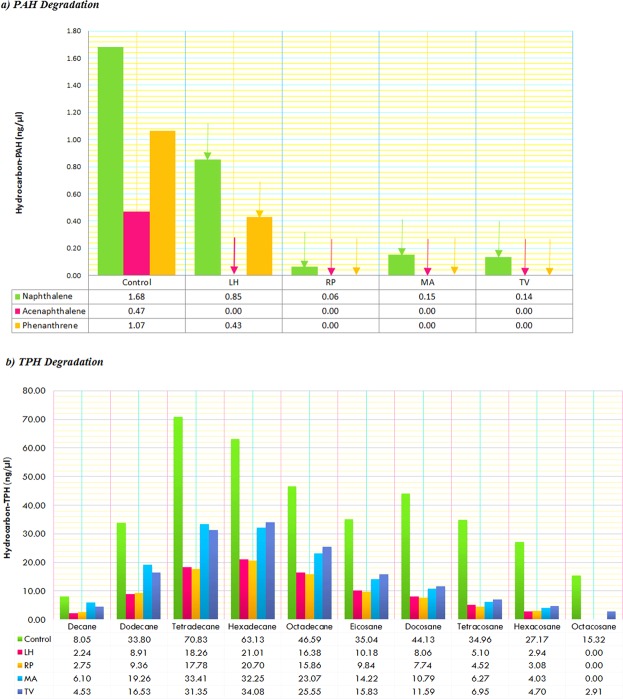
Utilization of various hydrocarbons (Total Petroleum Hydrocarbons and Poly Aromatic Hydrocarbons) by laboratory enrichments from various sites of oil pollution. LH, RP, MA and TV represents enrichments inoculated from oil contaminated sites at Light house, Royapuram, Marina and Thiruvanmiyur in the Chennai coast. Enriched samples from Royapuram and Light house showed maximum degradation in TPH and PAH.

Kinetics of population growth revealed that enrichments from different samples have varying growth patterns. Enrichment population inoculated with tarball yielded a growth pattern indicating alternate lag with slow and gradual increase. Enrichment with oil mousse showed a pattern of alternating peak and decline followed by a steady increase culminating in a stationary pattern between day 22 and day 30 (Supplementary Fig. S1). The hydrocarbon mineralisation by enrichment cultures was determined in Bushnell Hass (B-H) medium supplemented with heavy oil as the sole source of carbon. Hydrocarbon content analysis of the control flasks by mass spectra revealed a heterogeneous mixture with naphthalene (52%) as dominant portion of PAH and tetradecane (18%) to be the major portion of alkanes (Supplementary Figs S20 and 21). After 30 days of incubation, all evaluated enrichment cultures presented considerable hydrocarbon degradation potential although, enrichment inoculated with Royapuram samples where oil was contained in booms exhibited higher degradation potential with a reduction of 75% in TPH and 98% in PAH (Fig. 2). Spatial distribution of microbial communities from different polluted sites reflected their substrate specificities in enrichments, where octacosane was mineralized by all enrichments except Thiruvanmiyur (TV) and phenanthrene was utilized by enrichments from Royapuram (RP), Marina (MA) and Thiruvanmiyur (TV). Even though PAH represented the minor portion (0.84%) of the oil, wider and cumulative utilization of its components were detected in all enrichments investigated, specifically acenaphthalene was completely mineralized and >90% degradation of naphthalene was recorded in RP, MA and TV enrichments.

### Microbial communities in sediment, water and enrichment

**Figure 3:**
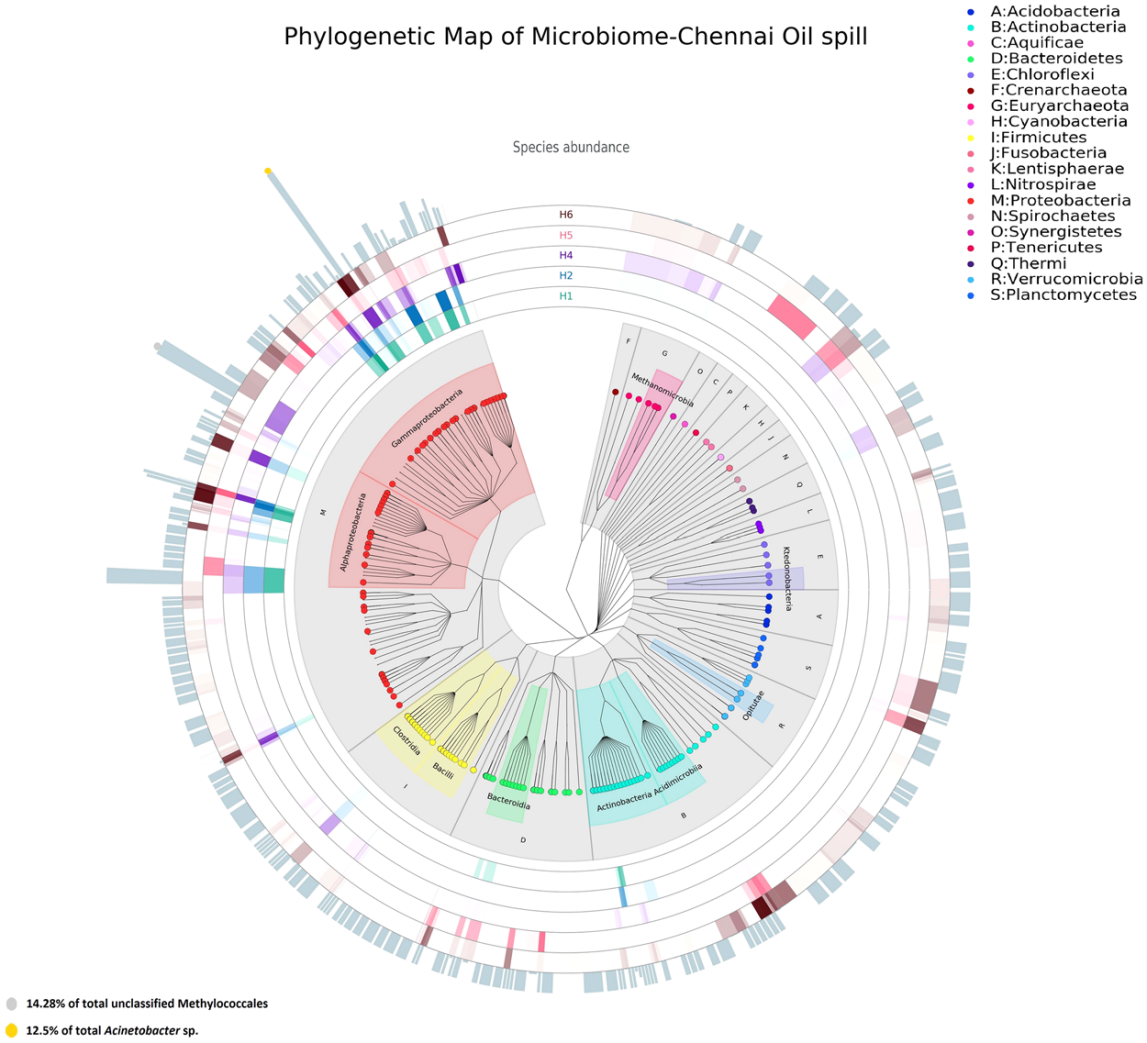
Map of diversity observed from oiled and non-oiled samples. Bacterial diversity was dominated by class gamma proteobacteria and alpha proteobacteria. Inner circle represent the phylogenetic tree and mid rings correspond to heat map of various genera distributed in different samples H1 (enrichment), H2 (oiled sediment), H4 (oiled water), H5 (non-oiled water) and H6 (non-oiled sediment). Bar plot on outside of the circle indicate the species abundance in all samples and height of the bar corresponds to absolute OTU count.

MiSeq High-throughput sequencing of 16S rRNA gene amplicons yielded 1502983 raw reads for bacteria and 597866 raw reads for fungi. Analysis of Rarefaction curve indicated that sequencing was able to capture the bacterial and fungal diversity efficiently (Supplementary Fig. S2). However, there were no amplicons obtained for H1 and H2 (enrichment and oil contaminated sediment) for fungi. OTUs generated could be identified into 387 genera, 215 families, 121 orders, 69 classes and 36 phyla for bacteria and 26 genera, 17 families, 15 orders, 10 classes and 3 phyla for fungi. Signature genera observed in oiled samples varied distinctly from that of non-oiled samples (Fig. 3) and the Mid-ring heat map of Fig. 3 demonstrates the distinctive distribution of various genera around different classes, indicating uniform distribution in non-oiled samples and clustered distribution in oiled samples. Taxonomic resolution of non-oiled samples (H5 and H6) detected higher levels of orders Thiotrichales, Stramenopiles, Rhodobacterales, Alteromonadales, Flavobacteriales and Pirullales (Supplementary Figs S3 and S4). Varying vulnerability of resident populations in sediment and in water was represented by congregation of exclusive taxa of orders Verrucomicrobiales, Acidimicrobiales, Saprospirales, Pirullales, and Thiotrichales that were detected only under non-oiled conditions. Although Gammaproteobacteria was the highest ranking class in all the samples, the percentage abundance showed a clear enrichment in sediment (36.15 to 78.21%, P ≤ 0.0001) as well as in water (29.5 to 68.52%, P ≤ 0.0001) after the spill event. Even though oil contaminated water was abundant in unclassified genera from the order Methylococcales, oiled sediment and enrichments presented abundance of characterized genera comprising *Acinetobacter*, *Alcanivorax*, *Shewanella*, *Hyphomonas* and *Thalassospira* (Supplementary Fig. S5). However, the primer combination used in this study for the detection of *alkB* gene typically reported in *Acinetobacter* sp. (Supplementary Table S3) had shown no PCR amplification.

Taxonomic affiliations of the fungal sequence reads were distributed into 56 species in all samples and similar to bacterial diversity, reduced evenness in species distribution was observed in oiled water compared to non-oiled water (Supplementary Fig. S7). Metagenomic sequences of fungi revealed the dominance (Supplementary Fig. S8) of unidentified group of fungi in the oiled water (83.75%) exhibiting the survival or enrichment of unknown taxa for which no taxonomic resolution further than kingdom level is available. However, in the non-oiled seawater, the unidentified fungus was represented by only 11.34% and was dominated by *Candida tropicalis* (32.62%). In general, other major groups of fungi detected include *Sporidiobolus ruineniae* (9.92%), *Saccharomyces cerevisiae* (8.5%) and uncultured Basidiomycota (8.5%). In contrast to fungal enhancement of specific taxonomic lineages in the water column under polluted conditions, lack of fungal amplicons in oiled sediment and enrichment is indicative of the susceptible nature of fungal communities to oil pollution.

The results of Principal Component Analysis (PCA) revealed clustering of oiled samples and enrichment, depicting the characteristic response of the microbial communities to oil pollution (Supplementary Fig. S9). Dissimilarities in distribution were also pronounced in diversity indices- Shannon index, evenness and richness. Similar to previous reports^22,24^ diversity indices were comparatively high for non-oiled samples and reduced in oiled samples. One of the major factors contributing to the reduction in oiled samples could be the dominance of *Acinetobacter* (52%, P ≤ 0.0001) in sediment and Methylococcales (30%, P ≤ 0.001) in water.

### Isolation and characterization

Isolation performed on B-H medium obtained 116 oil degrading isolates from heavy oil contaminated samples belonging to the classes Gammaproteobacteria, Alphaproteobacteria and Actinobacteria. The near complete 16S rRNA sequences (>1400 bp) of these isolates revealed that more than 60% were affiliated to order Oceanospirillales, Rhodospirillales and Micrococcales. Genus *Acinetobacter*, the dominant taxonomic group in metagenomic analysis, was represented only by 7% in the cultivable diversity and the culturables were predominated by *Thalassospira* (27%) and *Alcanivorax* (25%). Other major hydrocarbon degrading bacteria isolated include *Salinicola* sp., *Kocuria* sp. and *Martelella* sp. and interestingly were not represented by metagenomic community analysis. Species of *Gordonia* was represented by 0.002% (metagenome) in the oiled sediment and was represented by 5% of the cultivable bacteria. The near-complete 16S rRNA sequences of isolates and the retrieved sequences of previously reported hydrocarbon degraders were used to construct the phylogenetic tree (Supplementary Fig. S10). *Alcanivorax* (RS1, RS4, RS5, RS8 and RS18) isolated during the current study were affiliated to known hydrocarbon degrader *Alcanivorax dieselolei* PM07 (HM596594.1). Similarly, 8 isolates of *Thalassospira* were affiliated to three reported hydrocarbon degrading *Thalassospira* sp. (JX119044.1, AB265822.1 and EU239909.1) and two strains were related to *Acinetobacter calcoaceticus* ATCC 23055. Phenotypic characterization using biochemical analysis of isolates yielded 13 distinct groups (Supplementary Table S4). Strains of *Martelella* and *Salinicola* were found to have 2 phylotypes each differing in utilization of citrate, lysine, ornithine, glucose, lactose, arabinose and nitrate.

### Metabolic characterization and functional prediction of oil contaminated environment

Metagenomes were predicted by PICRUSt analysis to understand the metabolic potential of oil contaminated environment to identify its differential functional preferences. Among the categorized functions, metabolism was the most dominant class. In all evaluated cases, pronounced differences in xenobiotic degradation and metabolism were evident (Supplementary Figs S11–S14). Larger prevalence of gene annotations ordained to xenobiotic metabolism was detected in oiled seawater (7%) than non-oiled seawater (5%). Similarly, under oiled conditions in sediment, gene affiliations to this metabolism increased from 7% to 10%. In the laboratory enrichment (H1), a fairly substantial fraction (11%) of putative genes associated with catabolism of xenobiotics (Supplementary Fig. S15) contributed towards the total metabolism.

**Figure 4:**
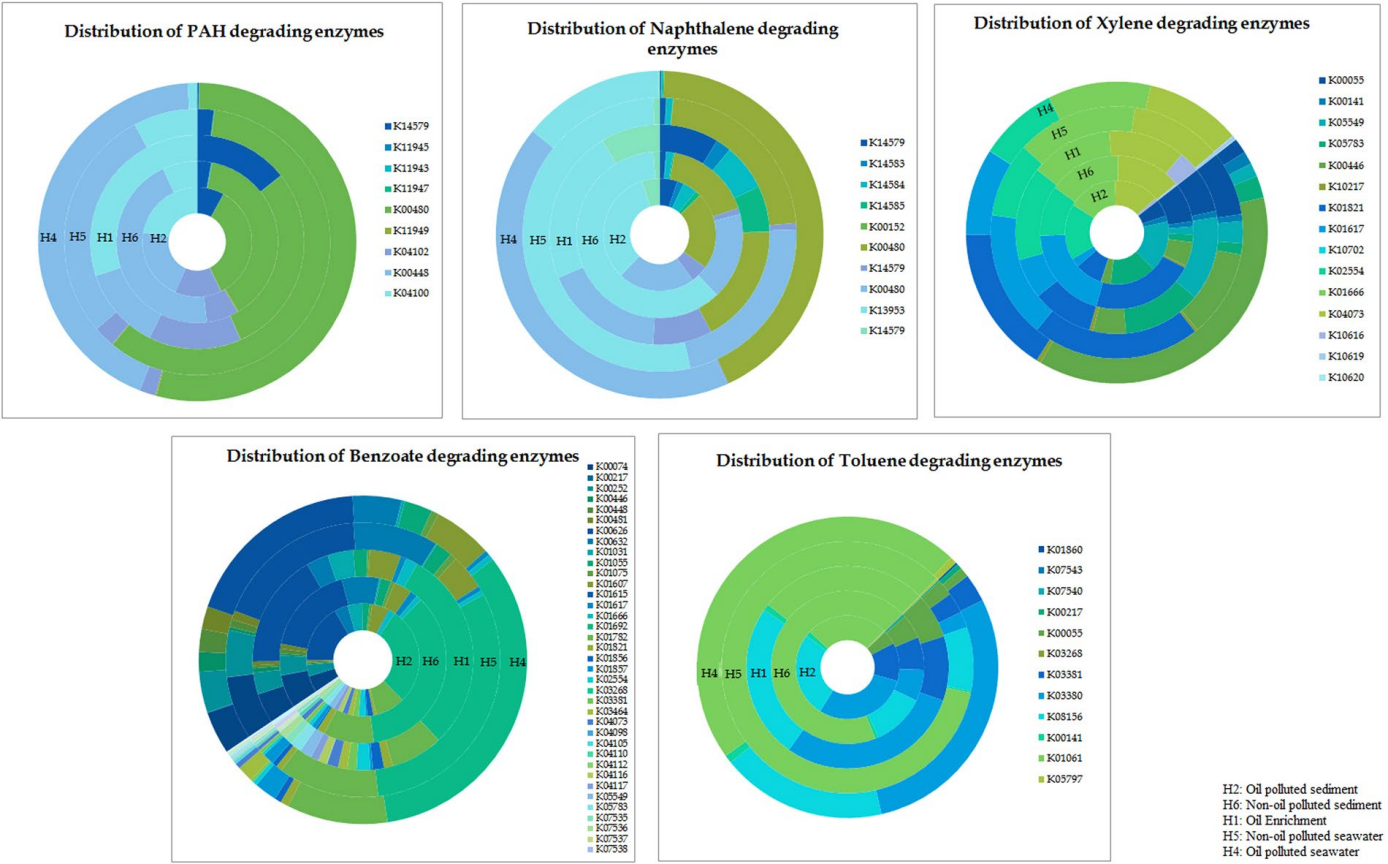
Relative distribution of enzymes involved in various hydrocarbon degradation (PAH, naphthalene, xylene, benzoate and toluene) in seawater, sediment and the enrichment. Different colours in the Pie chart represent various enzymes represented as KEGG Orthologs.

**Figure 5:**
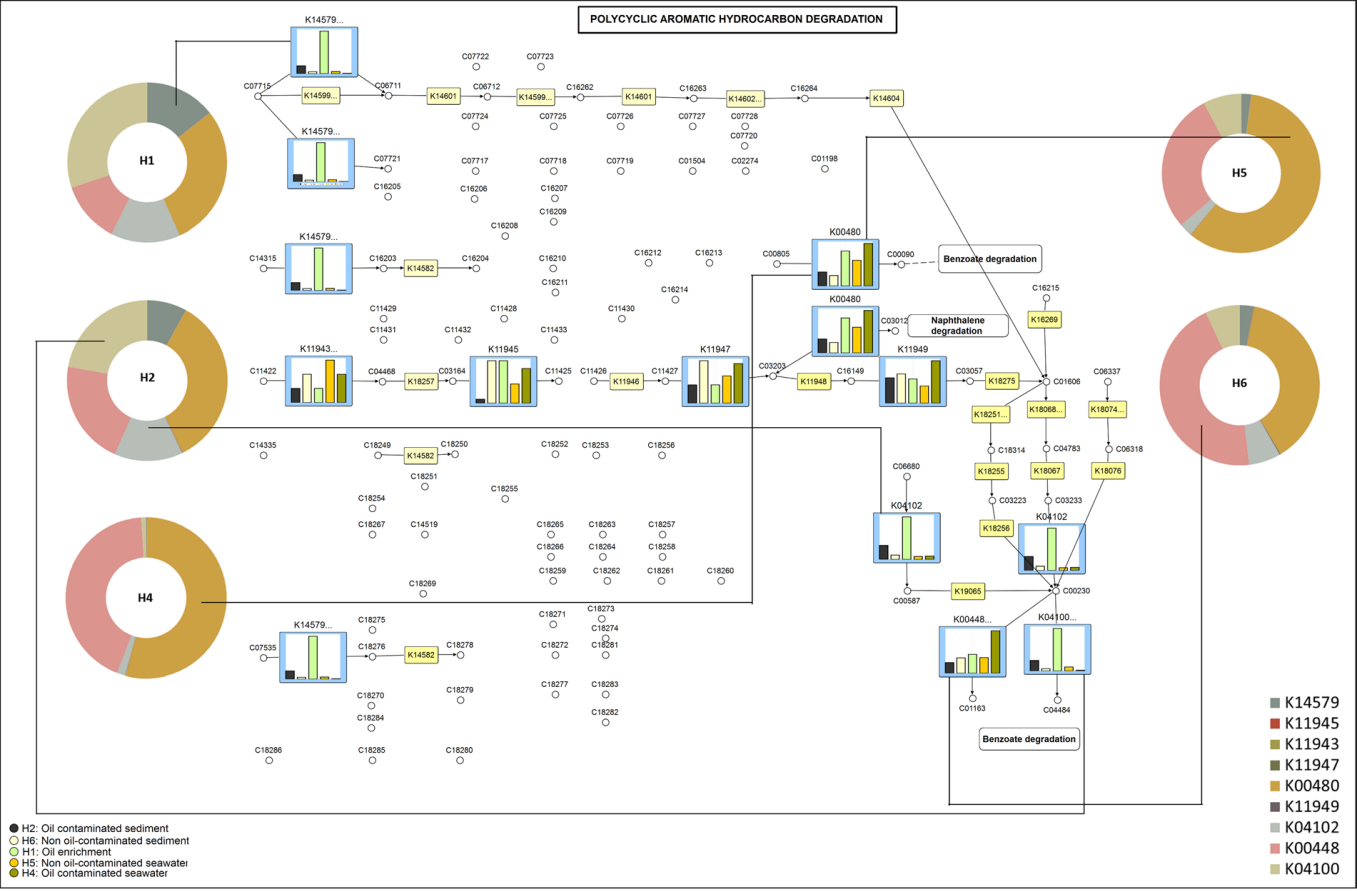
Potential Polycyclic Aromatic Hydrocarbon (PAH) degradation pathway in the investigated communities of different environmental matrices derived from 16S rDNA based KEGG orthologs. Donut charts indicating the abundance of enzymes in each sample and bar plots indicating the relative abundance of enzymes between samples. The oil contaminated/non-oil contaminated samples, enrichment and the KOs are represented using different colours. The KEGG pathway^75^ map 00624 is adapted here from http://www.kegg.jp/kegg/kegg1.html. and enriched with data using VANTED (V2.2.1).

**Figure 6:**
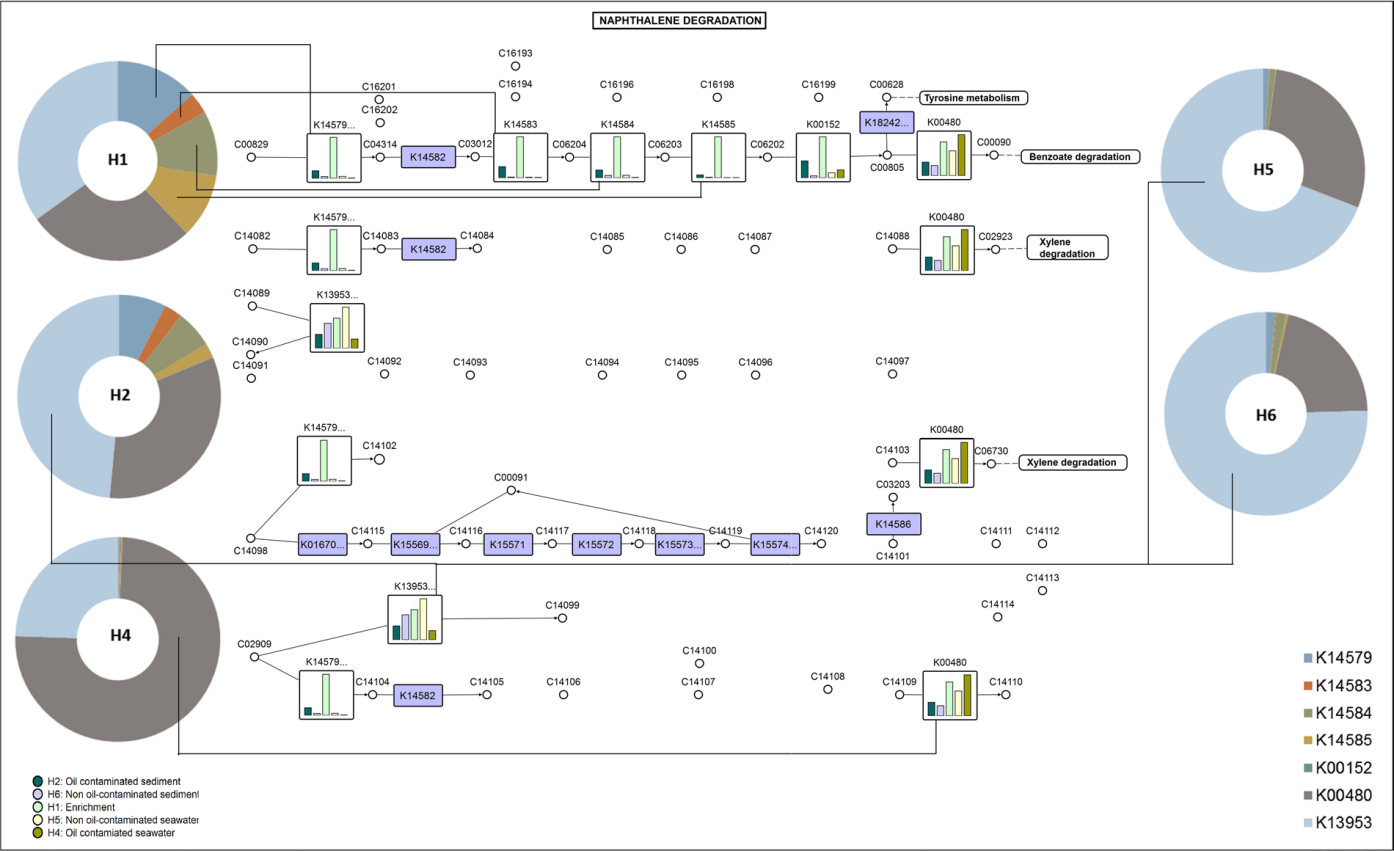
Potential naphthalene degradation pathway in the investigated communities of different environmental matrices derived from 16S rDNA based KEGG orthologs. Donut charts indicate the abundance of enzymes in each sample and bar plots indicate the relative abundance of enzymes between samples. The oil contaminated/non-oil contaminated samples, enrichment and the KOs are represented using different colours. The KEGG pathway^75^ map 00626 is adapted here from http://www.kegg.jp/kegg/kegg1.html. and enriched with data using VANTED (V2.2.1).

From the 16S rRNA metagenomic data, the proteins were predicted and classified as KEGG orthologs (KOs) with an identification of 6054 KOs across all samples. The ~780 Mb 16S rRNA sequences of H1, H2, H4, H5 and H6 contained 5 86377 encoded genes that could be associated with degradation of hydrocarbon. An array of metabolic genes involved in degradation pathways of PAH, naphthalene, benzoate, toluene and xylene were differentially abundant across all samples and their distribution is represented in Fig. 4. We mapped the enzymes detected in the samples to the KEGG pathways to understand the probable degradation of PAH (Fig. 5) and naphthalene (Fig. 6) with the respective OTU counts. The mapping of PAH pathway revealed the enrichment of oxidoreductases K00480, K14579, K04100, K00448 and lyase K04102 in oiled sediment and seawater. In the laboratory enrichment (H1), the genes encoding the enzymes K14579, K04102 and K04100 involved in PAH degradation were detected to be high. Non oiled sediment exhibited enhanced abundance of generalized oxidoreductases involved in carbohydrate and aldehyde metabolism (K11945 and K11947) however, a single oxidoreductase K11943 affiliated to PAH degradation was detected in non-oiled seawater.

Since naphthalene occupied the highest proportion of PAH in the current study, several genes coding for enzymes involved in its degradation were mapped in naphthalene degradation pathway. Post oil spill, high enrichment of oxidoreductases (K14579, K14583, K14584, K14585, K00152 and K00480) catabolizing naphthalene were recorded in both oiled seawater and sediment (Fig. 6). Additionally, mapping of KEGG pathway was also performed for benzoate degradation (45 enzymes), toluene degradation (12 enzymes) and xylene degradation (15 enzymes) as represented in the Supplementary Figs S16–S18. Apart from the hydrocarbon degradation, oiled sediment and seawater were found to have an enhancement of genes coding for degradation of limonene, pinene and geraniol, butanoate metabolism, beta alanine metabolism, carbon fixation and transcription factors. However, the genes associated with cell-cell communication, CAM and ion channels were detected to be fewer as a likely response to sudden influx of oil (Supplementary Fig. S19).

### Fatty acid profiling

In the oiled water, considerable enrichment of fatty acids C16:0, C18:1 w9c, C18:2 w6c and C18:0 were observed, among which C16:0, C18:1 w9c were previously reported as the dominant phospholipids enriched during oil plumes^11^. The oiled samples exhibited a considerable reduction in the C16:1 w7c DMA and C12:0 however, C19:0 cyclo w6c, C22:2 w6c, C22:0 10-methyl, C24:3 w3c and C24:0 were only detected in oiled water. Likewise, in the oiled sediment, C15:3 w3c was enriched and considerable reduction in fatty acids C12:0, C18:1 w9c, and 19:3 w3c was observed, suggestive of the distinct community responses to oil pollution in the water and sediment matrices. The eukaryotic marker C21:3 w3c was present both in oiled sediment (12.83%) and enrichment (11.55%) while marker C24:1 w7c was detected only in oiled sediment.

## Discussion

Microbial communities in sediment and water were dominated by the Gammaproteobacteria but their percentage abundance increased to 78.21% in sediment and 68.52% in seawater after the spill. Our results are consistent with previous reports in the enrichment of Gammaproteobacteria in response to influx of hydrocarbon^11,21^ however, the bloom (enriched) specific taxonomic group involved in oil degradation was found to be unique to the current spill i.e. *Acinetobacter* in sediment and Methylococcales in water. These unprecedented blooms draw attention towards the underexplored hydrocarbon degrading communities in tropical waters. The shift in community dynamics in sediment and water habitats caused by oil disturbance was reflected in concomitant reduction of evenness in taxa as represented in the heat map rings of diversity map (Fig. 3). In the heat map, intensities of genera were found to be high with a clustered distribution (represented above the class Gammaproteobacteria) in oil contaminated samples, in contrast to non-oiled samples with more or less equal distribution around various classes. This discriminant assemblage, owing to high relative abundance of core communities could be attributed to metabolic restrictions or competition for particular hydrocarbon substrate since the spilled heavy oil had characteristic high proportions of several medium chain alkanes^32–34^ and relatively moderate shares of naphthalene and phenanthrene in PAH.

From the metagenomic analysis, we narrowed down top 25 orders to highlight the signature characteristics of taxonomic distribution, which revealed the dominance of orders Pseudomonadales (in sediment) and Methylococcales (in water) after the spill event (Supplementary Fig. S6). *Acinetobacter* of order Pseudomonadales which was originally only 0.8% in the non-oiled sediment was represented by 52% of total OTU abundance in oiled sediment with a genomic assemblage of 46 distinct OTU ids. A few *Acinetobacter* spp. are previously reported to utilize petroleum, long chain alkanes and engine oil as sole carbon source, known to articulate physiological adaptation of biosurfactant production including emulsan and alasan aiding in hydrocarbon degradation^35,36^. Methylococcales represented 30% of the oiled water and interestingly not detected in the laboratory enrichments. This is because Methylococcales are obligate utilizers of methane under aerobic or microaerobic conditions and have been isolated from methane influx environments including marine waters and benthic sediments^37^. Removal of methane from medium-heavy oil by biodegradation is reported to be moderate^38^ and there is no direct evidence of oxidative methane biodegradation in marine environment post an oil spill. The abundance of Methylococcales in the present study, suggests the possibility of *in-situ* oxidative biodegradation of methane and metabolomic analysis was also indicative of this phenomenon, as high proportions of methane monooxygenase and methanol dehydrogenase were detected in the oiled water. In the laboratory enrichments, the taxonomic preferences in methane metabolism changes from methylotrophs to methanogens represented by archaea Methanosarcinales and Methanobacteriales, which could have enriched from oil contaminated sediment.

Oceanospirillales and Alteromonadales were observed to have high abundance in oil contaminated samples which represented a unique scenario where metabolic specialist Oceanospirillales containing hydrocarbonoclastic bacteria *Alcanivorax* (13.3%) and *Oleibacter* (7.2%) occupying same habitat with metabolic generalist Alteromonadales. Alteromonadales are reported to be highly adaptive to environmental stress^39^ and the order was represented by the genera *Shewanella*, unclassified Alteromonadaceae, *Pseudoidiomarina* and *Marinobacter* in the current study. The role of genera *Pseudomonas*, *Shewanella* and *Marinobacter* in oil degradation was reported from Arctic sea^40^ might indicate the global distribution of these oil degrading organisms. Although Oceanospirillales were found to have high abundance, differential assemblage of various genera of Oceanospirillales in the sediment and water were pronounced, wherein *Alcanivorax* was dominant in sediment and *Oleibacter* represented major genus in water. Moreover, considerable enrichments of fatty acids C16:0, C18:1 w9c in the oiled water, validates the previous report of these fatty acids as the dominant lipids in oceanospirillales^11^_._ Genus *Alcanivorax* has been reported as hydrocarbonoclastic bacteria from various marine environment around the world^41^ and in the present study, 20 distinct OTU ids were found to be affiliated to this genus. Interestingly, a few out of 20 OTU ids possibly signifying selective advantage, enriched up to 10000 folds under oiled conditions (OTU ids-82147, 818315, 25603 and 41476). Similarly, out of the 7 OTUs of *Oleibacter*, ids 1784974 and 160907 were enriched up to 9000 folds in the oiled seawater indicating unique footprints of genomic diversity could be responsible for site-specific degradations.

Several orders including Rhodobacterales, Flavobacteriales, Bacillales, Clostridiales and Rhizobiales were found in high abundance not only in oiled and non-oiled samples but also in the laboratory enrichments suggesting degradation potential of underexplored taxonomical groups. Taxonomic blooms of Flavobacteriaceae and Rhodobacteriaceae were previously reported to be responsible for scavenging soluble hydrocarbons^19^. This iterates the degradation potential of native communities in different habitats and also indicates the presence of hydrocarbon degraders that flourish independent of receiving an oil influx. However, vulnerability to different environmental stress varies among taxonomic lineages where 4 orders from non-oiled sediment and 8 orders from non-oiled water have vanished following the spill event from the top 25 orders (Supplementary Fig. S6). Analysis between the oil contaminated samples, revealed the exclusive assemblages of some taxonomic groups to be site- and spill- specific, the orders such as Flavobacteriales, Solibacterales and Pedospirales were only found in sediment while Synechococcales and Rickettsiales were detected only in water. Nitrospirales, which are reported to be nitrifying marine bacteria detected in the non-oil contaminated water, have been replaced by Nitrosospirales, known ammonia oxidizers in the oiled water.

Hydrocarbon content analysis revealed high proportions of mid-length alkanes such as tetradecane (70.82 ng/µl), hexadecane (63.12 ng/µl) and octadecane (46.58 ng/µl) among the 10 alkanes detected in the sample. Dynamic changes in relative abundance of genera that bloomed in the oiled conditions were in concurrence with compositional distinctiveness of the spilled oil. For example, Tetradecane degradation efficacy of many species of *Acinetobacter* have been reported to be high and specifically, *Acinetobacter venetianus* was reported to prefer tetradecane as the optimal carbon source out of C_10_ to C_25_ alkanes^39,40^. Moreover, the present study reports for first time the blooms of genus *Acinetobacter* in marine sediments and order Methylococcales in seawater in response to an oil spill. Hexadecane and Octacosane degradation was reported from taxonomic groups such as *Pseudomonas*, *Flavobacterium*, *Alteromonas* and Oceanospirillales which were all found to be enriched in the current study^41–45^. Components detected in PAH analysis were naphthalene, acenaphthalene and phenanthrene, of which naphathalene was found to occupy largest proportion. Genera such as *Thalassospira* and *Pseudomonas*^46–51^ have been reported to be specifically affiliated to naphthalene degradation which was also detected to be enriched (P ≤ 0.001) in the oiled samples.

Similar to metagenomic analysis, phylogenetic identification of isolates revealed co-existence of generalist bacteria with specialist and the later was found to be affiliated to previously reported hydrocarbon degrading bacteria such as *Alcanivorax dieseloli* and *Thalassospira*. Rodriques *et al*. 2015 recorded such shared habitat existence in the beach communities during deep water horizon oil spill and our result extrapolates on their findings. The study suggests the response and survival of these organisms on complex habitats in response to multi-substrate disturbances that depend upon niche specialization owing to their selective adaptations. The phenotypic characterization further extends the proof of above hyphothesis where *Alcanivorax* and *Thalassospira* were unable to metabolize glucose, lactose, adonitol, arabinose, sorbitol, citrate, lysine and ornithine while the generalist groups (*Kocuria* sp., *Martelella* sp., *Labrenzia* sp., *Pseudomonas* sp., *Ochrobactrum* sp., *Gordonia* sp., *Salinicola* sp. and *Microbacterium* sp.) were able to utilize them (Supplementary Table S4). Out of the 46 different OTU IDs of *Acinetobacter* sp. retrieved from the NGS analysis, the species representing the OTU ID 343318, was successfully obtained as pure culture and deposited in GenBank under the accession number MF661951.1. Although *Acinetobacter* is considered to be metabolically generalist group, the phenotypic characterization specifically highlights the inability to utilize simple sugars, suggesting that some species of this genus could be obligate hydrocarbonoclastic in nature. We propose a re-evaluation of degradation potential of the genus *Acinetobacter* since it might be contributing to a greater extent in hydrocarbon clean up in marine environment than previously thought.

The current study revealed enrichment of unclassified fungi from 14 to 83% in the oiled water, and the role of these uncharacterized fungi in degradation of hydrocarbon are unknown. Several fungal genera *Cochilobolus*, *Sclerotina*, *Neosartoria*, *Mycosphaerella*, *Trichosporon*, *Penicillium*, *Auricularia* and *Teratosphaeria* were only found in oil contaminated seawater, of which *Cochilobolus*, *Neosartoria*, *Penicillium* and *Trichosporon* have been previously reported for hydrocarbon degradation potential^25,52–55^. Interestingly, in the oiled seawater, *Candida* and *Rhodotorula* reduced in abundance by 33 to 1% and 0.7 to null respectively, even though these genera are reported to be hydrocarbon degraders from terrestrial environment^53^. Non-oiled sediment represented a total of 19 fungal species from the metagenomic analysis. These were however non-amplified in the oiled samples or enrichments indicating the possible vulnerability of these taxonomic groups to toxic effects of spilled hydrocarbons. Our data reiterates the importance of elaborate studies on potential of hydrocarbon degrading fungi and vulnerability of fungal communities to the toxic effects of such pollutants in the marine environment.

Results of PICRUSt analysis was suggestive of scenarios that the structurally dissimilar communities could manifest similar trends of outstretched functions, supportive of the functional redundancy^56^ to microbial communities. However, specific functions such as cell to cell communication, xenobiotic biodegradation, environmental adaptation and information processing that includes membrane transport, signalling molecules and their interactions, were possibly affected by community diversity and composition. Understanding such microbial community and compositional variations with their functional shift in response to oil pollution would help devise appropriate strategies for *in situ* bioremediation of hydrocarbon^57–59^. In the current study, the metabolic genes coding for naphthalene 1, 2-dioxygenase involved in both PAH and naphthalene degradation were mainly affiliated to Sphingomonadaceae, Erythrobacteraceae, and Comamonadaceae. Among these families, Erythrobacteraceae and Comamonadaceae were not represented in the top 25 predominant orders, indicative of their role in utilizing the minor portion (PAH) of the heavy oil. Family Sphingomonadaceae, though present in both oiled and non-oiled samples, enhancement was dominant in oiled sediment and the species belonging to this family are reported to be capable of degrading a wide range of PAHs due to greater catabolic versatility^60,61^.

Although it demands further confirmation by shotgun sequencing, several oxidoreductases with broad substrate specificity that include alkane 1-monooxygenase and phenol 2-monooxygenase were predicted to be enriched in both oiled seawater and sediment, substantiating the earlier report^7^. Fatty acid CoA ligase fadD32 reported to be required for the coupled import and activation of exogenous long-chain fatty acids^62^ were enriched in oiled sediment and laboratory enrichment. The role of dehydrogenases in converting intermediates of hydrocarbon degradation and competitive advantages of ion channels and transport proteins in nutrient limiting environment has been established^16,63^. In the current study, alcohol and aldehyde dehydrogenases, Major Facilitator Superfamily (MFS) transporter system and benzoate membrane protein were also found to be enriched in oiled sediment and seawater.

In conclusion, differential distribution of bacterial and fungal communities was observed in sediment and water ecosystems post the oil spill event. However, several common taxonomic orders that are known generalist hydrocarbon degraders, were observed in abundance in both the oil contaminated matrices, indicate the minimal distribution of these degraders despite the polluted/unpolluted nature of the habitats. Metabolomic analysis revealed the enhancement of gene copy numbers possibly involved in degradation of major components of spilled heavy oil in sediment and seawater. Furthermore, the current study depicted distinct difference in bloom composition of oil spill in a tropical region in contrast to previously reported blooms from sub-tropical and temperate regions around the world indicating varied existence of competitive advantages of native population. High proliferation of *Acinetobacter* sp. after the oil spill, which is proposed to be re-evaluated for its hydrocarbon degradation potential in this study, might play a key role in natural attenuation of hydrocarbon in the tropical beaches. Although the study demands further research, metagenomic and metabolomic knowledge complemented with successful enrichment of efficient degraders under *in vitro* conditions will irrefutably contribute to the possibilities of developing nature identical solution and biomitigation intervention strategies that could be applied to unique environment.

## Methods

### Sample description and physicochemical analysis

Major portion of the oil slick was trapped near the groins of Royapuram and a substantial part dispersed towards south of Chennai coast (Fig. 1). The samples were collected on February 1, 2017, four days after the report of spill event. The leaked heavy oil, oil mousse, contaminated seawater and sediment samples were collected at Royapuram (13°113.67N; 80°191.43E) followed by Marina (13°340.19N; 80°1714.67E), Light House (13°29.69N; 80°1651.95E) and Thiruvanmiyur (12°5829.51N; 80°161.35E) towards south. The heavy oil was sampled from the surface of trapped oil slick near the groins and the contaminated water samples were collected from subsurface sea in 1 litre Nalgene bottles. Post the spill event, supratidal zone of southern coast of Chennai was characterized by the oil mousse and tarballs that washed ashore. Samples from these contaminated beach sediments (1 kg) were collected using PVC tubes at a depth of 15–20 cm.

For the comparative analysis of microbial community, non-oiled water and sediment were collected away from the slick area where no visible signs of oil contamination were observed. All the samples were processed within 24 hours for enrichment and the remaining subsamples were stored at −20 °C for further analysis. To better define physicochemical properties of the contaminated sites, variables including temperature, pH, salinity, dissolved oxygen were also recorded (Hydrolab MS5).

### Enrichment, growth study and hydrocarbon content analysis

The oil contaminated samples were enriched in modified Bushnell Hass (B-H) medium supplemented with 3% NaCl and 1% heavy oil (collected from spilled site) as sole carbon source^64^. Aliquots of samples were added to 100 ml of media and incubated at 28 °C in rotary shaker at 180 rpm for 30 days. Subsequent sub-culturing was carried out by adding 5 ml of enriched culture in to the fresh medium. After a series of three sub-culturing, matured inoculums were spread plated on to B-H agar and phenotypically distinct colonies were purified. The isolates exhibiting pronounced growth on heavy oil were selected and stored in glycerol stocks at −20 °C for further characterization.

The hydrocarbon content of the spilled heavy oil was determined using GC-MS (Agilent, 6890GC) for TPH and PAH. The composition of detected compounds is represented in Supplementary Table 5 and the same heavy oil was used for the growth and degradation assays. To deduce the growth curve and peak degradation activity of the enrichment cultures, 5 ml of seed inoculum was dispensed into B-H media supplemented with 1% heavy oil and incubated as described earlier (all experiments were carried out in triplicates). The growth was assessed every 24 hours for 30 days by measuring the turbidity (OD_600_ nm) using a UV visible spectrophotometer (Agilent G9821A). The hydrocarbon degradation activity of enrichment cultures was determined at 30^th^ day of incubation. The entire content of the flasks was extracted for Poly Aromatic Hydrocarbons (PAH) and Total Petroleum Hydrocarbons (TPH) using dichloromethane (DCM) at a ratio of 2:1 by aqueous two phase extraction. The DCM fraction was collected and the aqueous phase was re-extracted for an additional 3 times. The collected fractions were then dehydrated using magnesium sulfate (MgSO_4_) and concentrated by rotary evaporator to a final volume of 10 ml. Extracted hydrocarbons were analyzed by GC-MS (Agilent, 6890GC) equipped with DB-5 capillary column (30 m × 0.25 mm; 0.25 µm film thickness). Nitrogen gas was applied as a carrier gas with a flow rate of 1 ml/min. The column oven temperature was set at 40 °C with a hold time of 1 min and subsequently increased to 300 °C with a ramp of 10 °C/min with a final hold of 31 min. Both the injector and detector temperatures were set at 280 °C. Degradation efficiency was calculated by comparing peak areas of hydrocarbon^65^.

### Identification of bacterial isolates

The DNA of bacterial cultures was extracted using a DNeasy UltraClean Microbial Kit (Qiagen) and amplification of 16S rRNA gene (Supplementary Table S3) was performed^66^. The PCR amplicons were examined using agarose gel electrophoresis, purified and sequenced by Sanger sequencing method. The obtained sequences were checked for anomalies using Pintail^67^ and used for Basic Local Alignment Search Tool (BLAST) analysis against NR database in the National Centre for Biotechnology Information (NCBI) GenBank. Phylogenetic trees based on 16S rRNA gene sequences were constructed using the MEGA 7.0 software package^68^. Pairwise and multiple alignment were performed using CLUSTALW with all parameters set at default values and bootstrap calculation of 1000 runs.

### MiSeq sequencing

Total genomic DNA was extracted from seawater, sediment and enrichment using PowerSoil^®^ DNA Isolation kit (MoBio) according to manufacturer’s instructions. The quality of the extracted DNA was determined by agarose gel electrophoresis and in NanoDrop by determining A260/280 ratio. Amplification of 16S rDNA gene targeting V3-V4 region specific for bacteria and ITS region specific for fungi were carried out and amplicon libraries were prepared using Nextera XT index kit. The amplicon libraries generated were purified by 1x AMpureXP beads and quantified using Qubit Fluorometer (Thermofisher). Quality of the purified amplicons were checked using 4200 Tape Station system (Agilent Technologies) by D1000 screen tape followed by sequencing at Illumina Miseq platform (Eurofins Genomics). Sequences were clustered and Operational Taxonomic Units (OTUs) were generated based on ≥97% sequence similarities using Qiime^69^ and aligned with PyNAST^70^ using Greengenes database. Ribosomal Database Project (RDP) at a minimum confidence of 0.85 was used for assigning taxonomy to the OTUs. The taxonomic analysis of the metagenomic reads were performed using the software MEGAN CE^71^. Principal Component Analysis (PCA) was carried out to explore bacterial community shifts between oiled and non-oiled samples according to their OTU_97_ composition. The metagenomes and the annotated phylogenies were visualized using GraPhlAn^72^.

### PICRUSt analysis

Functional annotations of the assigned OTUs were performed using PICRUSt software^73^. The closed reference OTUs were picked by Qiime^69^ and sequences were aligned using Greengene database. After normalizing the gene copy numbers, the generated metadata was converted to BIOM table and the PICRUSt metabolic predictions were carried out at 97% similarity level. The relative abundance of functional categories as Kyoto Encyclopaedia of Genes and Genomes (KEGG) pathways were generated from OTU table of assigned taxa and were integrated and interpreted in KEGG pathways^74,75^. The mapped enzymes and the metabolite data were visualized using the software tool VANTED (version V2.2.1)^76^.

### PLFA analysis

PLFAs were extracted from sediment and water samples by sonication for 10 min after the addition of single phase Bligh-Dyer mixture^77^ of chloroform: methanol: phosphate buffer (1:2:0.8). Lipid classes were separated by solid phase extraction (SPE) using 96 well SPE plate containing 50 mg of silica per well and conditioned with methanol (3 × 1 ml) followed by chloroform (3 × 1 ml). Samples were dissolved in 1 ml chloroform, transferred to SPE plate and allowed to pass through the column. Phospholipids were eluted with 0.5 ml of methanol: chloroform: H_2_O mixture (5:5:1) and the solution was evaporated with N_2_. Following the addition of 0.2 ml transesterification reagent (25 ml toluene: 75 ml methanol: 0.561 g KOH), samples were dried with N_2_ and phospholipids were dissolved in 75 µl of hexane. Lipid content was analyzed using a Gas Chromatograph by ChemiStation (Agilent 7820 A) and Sherlock software (MIDI Inc.).

### Accession numbers-SRA site

High- throughput 16S rRNA sequencing data generated in the current study was deposited in NCBI Sequence Read Archive (SRA) under accession numbers SRS2715247, SRS2715018, SRS2715017, SRS2715016 and SRS2714032. Metagenomes are bundled under the NCBI Bioproject ID PRJNA419793. Hydrocarbon degrading isolates were deposited under the accession numbers MF973212-MF973221, MF972870-MF972883 and MF661946-MF661957.

## Supplementary information

Supplementary tables and figures

## Data Availability

All data generated or analysed during this study are included in this published article (and its Supplementary Information files) and raw datasets are available from the corresponding author on reasonable request.
