# Supplementary material for: Oil-Spill Triggered Shift in Indigenous Microbial Structure and Functional Dynamics in Different Marine Environmental Matrices
Source: Sci Rep. 2019 Feb 4;9:1354. doi: 10.1038/s41598-018-37903-x (PMC6361881; doi:10.1038/s41598-018-37903-x)
Supplement: Supplementary file 1 — Supplementary tables and figures [file 41598_2018_37903_MOESM1_ESM.pdf]

Scientific Reports

Supporting Information

Oil-Spill Triggered Shift in Indigenous Microbial Structure and Functional Dynamics in Different Marine Environmental Matrices

Neethu C Sainjan*, Saravanakumar Chinnasamy*, Purvaja Ramachandran, Robin RS and Ramesh Ramachandran

[1]National Centre for Sustainable Coastal Management (NCSCM), Ministry of Environment, Forest and Climate Change (MoEFCC), Chennai-600025, India.

Correspondence and request for materials should be addressed to RR (email: rramesh@ncscm.res.in) and PR (email:purvaja.ramachandran@gmail.com) and CS (email: cpshravan@gmail.com).

[*]These Authors contributed equally to this work.

Running title: Oil-spill triggered microbial dynamics

Microbial diversity analysis

Culture-dependent and metagenomic methods were performed to elucidate the microbial community structure in each habitat (Oil and Non-oil contaminated environment). The biochemical characterization of the isolated strains is represented in the supplementary table 4. The phylogenetic tree of the isolated strains was generated using MEGA (Version 7.0.20) with a bootstrap calculation of 1000runs. The phylogenetic affiliation of these isolates with the previously reported hydrocarbon degrading strains is represented in the supplementary figure 10. The metagenomic data was analyzed using interactive microbiome analysis tool MEGAN CE and the distinct microbial community structure observed between different habitats are represented in supplementary figure 3 to 5. MEGAN CE was also used to represent the phylogenetic tree of fungi with comparative analysis of relative abundance distributed in to various genera in different samples (supplementary figure 7). Pearsons-correlation ecological index with a stress level of 0.48 was used to generate Principal Coordinates Analysis (PCoA) to analyze the distribution of bacteria and fungi in different samples which is represented in supplementary figure 9.

Functional characterization

The metagenomic data was analyzed using PICRUSt software to illustrate the functional diversity and metabolic potential of microbial communities represented in supplementary figure 11 to15. Metabolic profiling tools, KEGG mapper and VANTED (Version V2.2.1) were used for mapping, integration and visualization of catabolic network of different degradation pathways represented in supplementary figure 16 to 18.

Supplementary Table 1: Observed physicochemical parameters at sampling locations of Chennai oil spill

Location	Latitude	Longitude	Air temp.	Wind Speed	Relative humidity	Heat Index	Barometric Pressure	Water temp.	pH	ORP	Salinity	D.O saturation	D.O
**Royapuram**	13°11'3.17"N	80°19'1.57"E	30.4	0.7	50.8	34.7	14.84	24.60	7.83	416.2	33.25	96.52	7.94
**North Marina**	13° 3'45.41"N	80°17'16.27"E	32.6	0.4	52.4	36.4	14.78	27.91	7.76	410	32.76	103.45	6.68
**Light House**	13° 2'21.51"N	80°16'53.54"E	32.6	0.4	52.4	36.4	14.78	27.91	7.76	410	32.76	103.45	6.68
**Thiruvanmiyur**	12°58'32.37"N	80°16'1.53"E	32.3	0.3	49.1	36.9	14.78	27.26	7.74	389.32	32.67	85.41	5.64

Supplementary Table 2: Percentage composition of C, H, N pre and post oil spill event

Sampling	N (%)	C (%)	H (%)	S (%)	C/N
**After Oil spill (Avg ± Std)**	0.06±0.01	3.93±0.08	1.79±0.14	1.63±0.21	63.15±9.35
**Before Oil spill (Avg ± Std)**	0.06±0.01	1.00±0.10	0.45±0.09	0.21±0.04	16.80±2.60

Supplementary Table 3: Primers used for amplification of various genes in this study

Primer	Domain	Sequence 5ˡ to 3ˡ	Reference
**fD1**	16S rDNA- Culturable bacteria[1]	AGAGTTTGATCCTGGCTCAG	Weisburg *et al.*, 1990
**rP2**	16S rDNA- Culturable bacteria[1]	ACGGCTACCTTGTTACGACTT	
**alk F**	*alk* B gene-hydrocarbon degradation[2]	GCI CAI AR ITI RKI CAY AA	Kuhn *et al.*, 2009
**alk R**	*alk* B gene-hydrocarbon degradation[2]	GCI TGI TGI TCI SWR TGI CGY TG	
**16S-f**	16S rDNA-Metagenome[3]	GCCTACGGGNGGCWGCAG	Klindworth *et al.*, 2012
**16S- r**	16S rDNA-Metagenome[3]	ACTACHVGGGTATCTAATCC	
**ITS -f**	18S rDNA-Metagenome[4]	GCATCGATGAAGAACGCAGC	White *et al.*, 1990
**ITS-r**	18S rDNA-Metagenome[4]	TCCTCCGCTTATTGATATGC	

Supplementary Table 4: Biochemical characterization of hydrocarbon degrading bacterial isolates enriched on BH media

Selected Isolates	Citrate Utilization	Lysine	Ornithine	Urease	TDA	Nitrate reduction	H2S production	Glucose	Adonitol	Lactose	Arabinose	Sorbitol	Accession no:
***Alcanivorax dieseloli* RS1**	-	-	-	-	-	-	-	-	-	-	-	-	MF661952.1
***Alcanivorax* sp . RS4**	-	-	-	-	-	-	-	-	-	-	-	-	MF973221.1
***Alcanivorax* sp. RS5**	-	-	-	-	-	-	-	-	-	-	-	-	MF661954.1
***Labrenzia* sp. RS15**	-	-	-	-	-	-	-	+	-	-	+	-	MF973215.1
***Thalassospira* sp. RS7**	-	-	-	-	-	-	-	-	-	-	-	-	MF973220.1
***Thalassospira* sp. RS10**	-	-	-	-	-	-	-	-	-	-	-	-	MF973219.1
***Gordonia* sp. H21**	+	-	+	-	-	+	-	+	-	-	-	-	MF972883.1
***Acinetobacter* sp. RC 33**	-	-	-	-	-	-	-	-	-	-	-	-	MF661951.1
***Thalassospira* sp. RC 7**	-	-	-	-	-	-	-	-	-	-	-	-	MF661949.1
***Kocuria* sp.RC38**	-	+	-	-	-	-	-	+	-	-	-	-	MF661957.1
***Martelella* sp.RS23**	-	-	+	-	-	-	-	-	-	-	-	-	MF661946.1
***Martelella endophytica* RC 49**	+	-	-	-	-	+	-	+	-	-	-	-	MF973217.1
***Thalassospira* sp. RS 3**	-	-	-	-	-	-	-	-	-	-	-	-	MF661953.1
***Salinicola* sp.LH4**	-	+	-	-	-	-	-	+	-	+	-	-	MF972878.1
***Salinicola* sp. VLH3**	+	-	-	-	-	-	-	+	-	-	+	-	MF972881.1
Ochrobactrum oryzae RC20	-	-	-	-	-	+	-	-	-	-	-	-	MF972880.1
***Pseudomonas aeruginosa* RC 22**	+	-	-	-	-	-	+	+	-	-	-	-	MF972873.1
***Microbacterium* sp. H22**	+	-	+	-	-	-	-	+	-	-	-	+	MF972875.1

Supplementary Table 5: Composition of spilled heavy oil as determined by GC-MS analysis

S.No	Composition	Amount (ng/µl)
**Total Petroleum Hydrocarbon (TPH)**		
**1.**	Decane	8.048
**2.**	Dodecane	33.804
**3.**	Tetradecane	70.825
**4.**	Hexadecane	63.126
**5.**	Octadecane	46.583
**6.**	Eicosane	35.044
**7.**	Docosane	44.130
**8.**	Tetracosane	34.964
**9.**	Hexacosane	27.167
**10.**	Octacosane	15.323
**Polycyclic Aromatic Hydrocarbon (PAH)**		
**11.**	Naphthalene	1.683
**12.**	Acenaphthalene	0.471
**13.**	Phenanthrene	1.065

Supplementary Figure 1: Growth kinetics of enrichments from different oil polluted samples

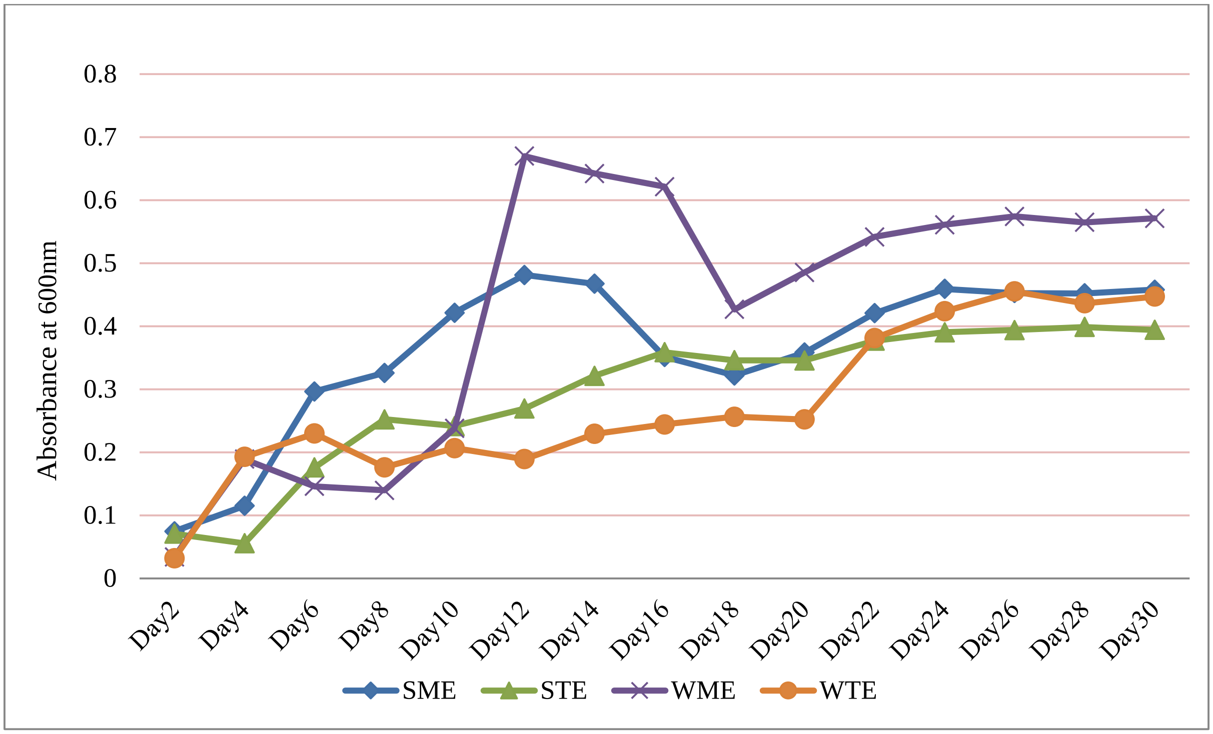

SME: Sediment Mousse Enrichment

STE: Sediment Tar-ball Enrichment

WME: Water Mousse Enrichment

WTE: Water Tar-ball Enrichment

Supplementary Figure 2: Rarefaction curve of bacterial and fungal sequences

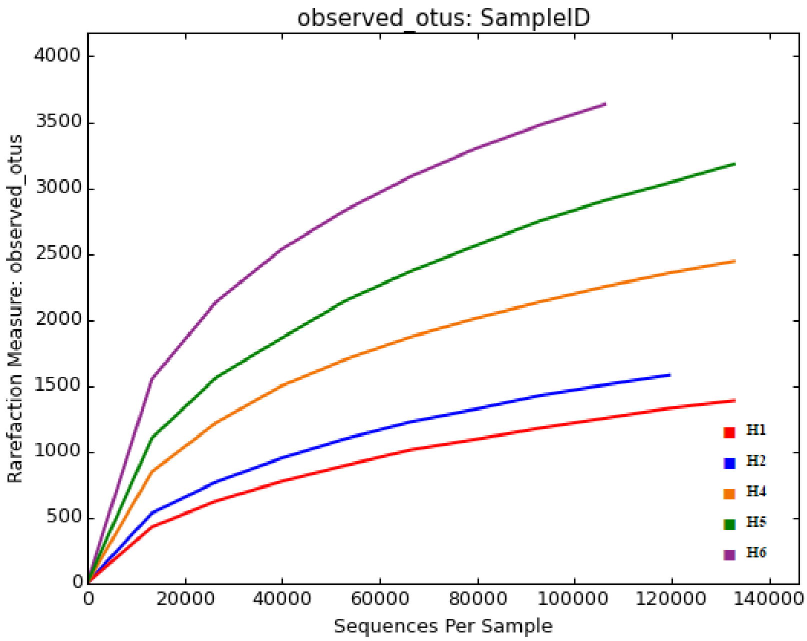

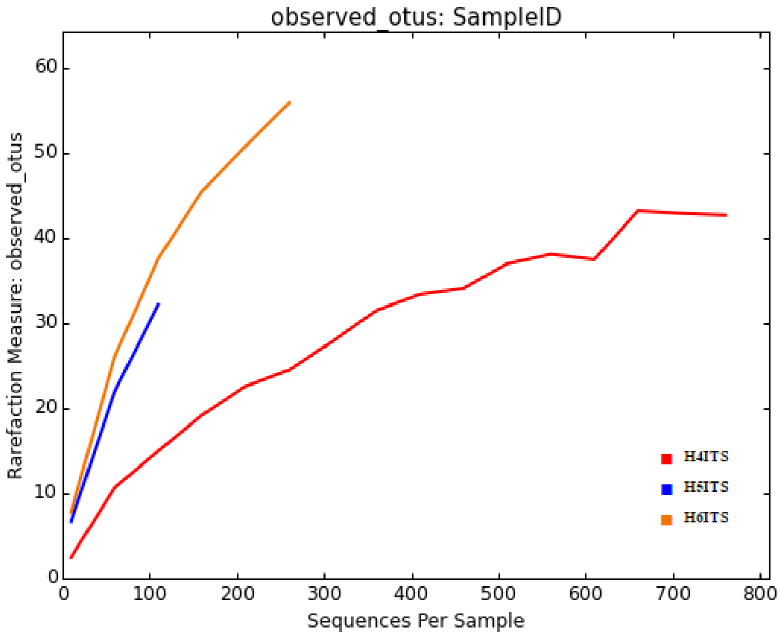

H1- Enrichment; H2- Oil contaminated sediment; H4- Oil contaminated water; H5- Non-oil contaminated water; H6- Non- oil contaminated sediment

Supplementary Figure 3: Bacterial abundance at Genus level in non-oil contaminated and oil contaminated seawater (H5, H4)

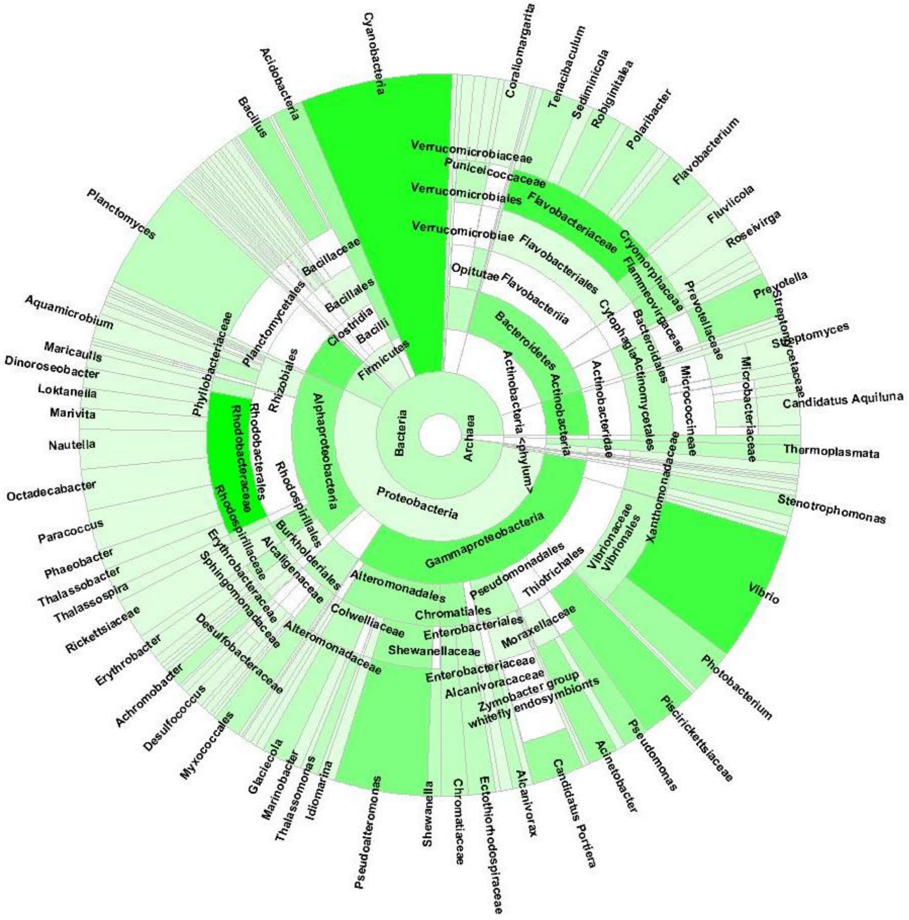

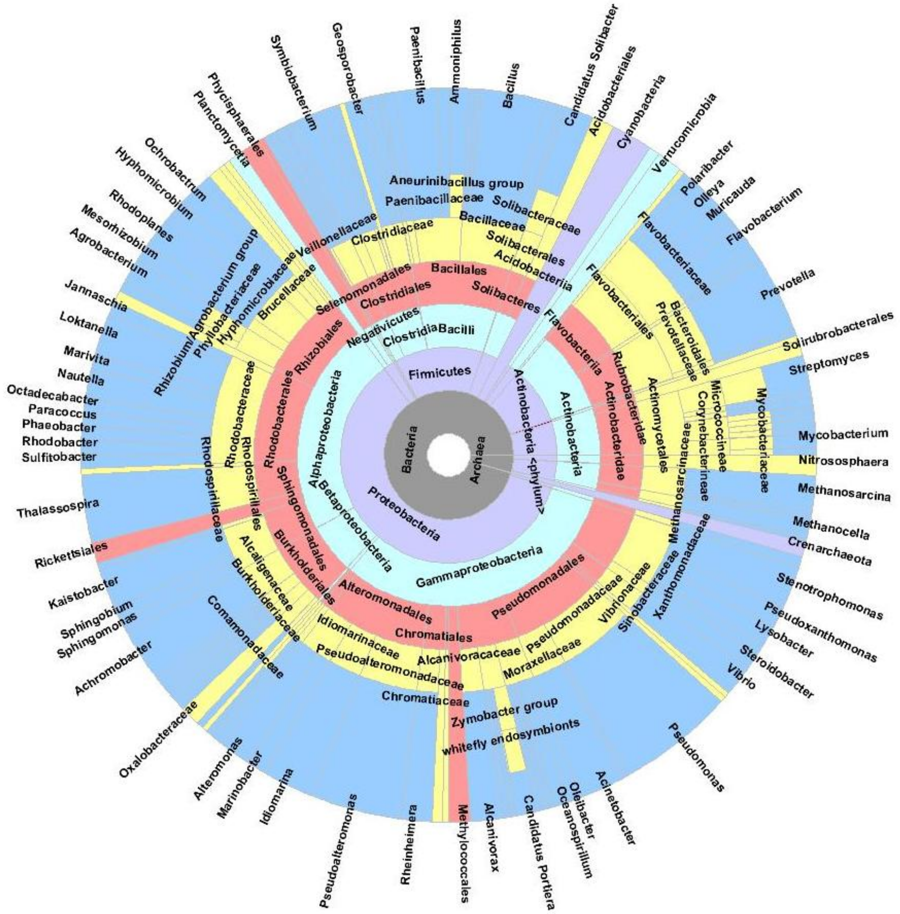

Non-oil contaminated seawater (H5)

Oil contaminated seawater (H4)

Supplementary Figure 4: Bacterial abundance at Genus level in non-oil contaminated and oil contaminated sediment (H6, H2)

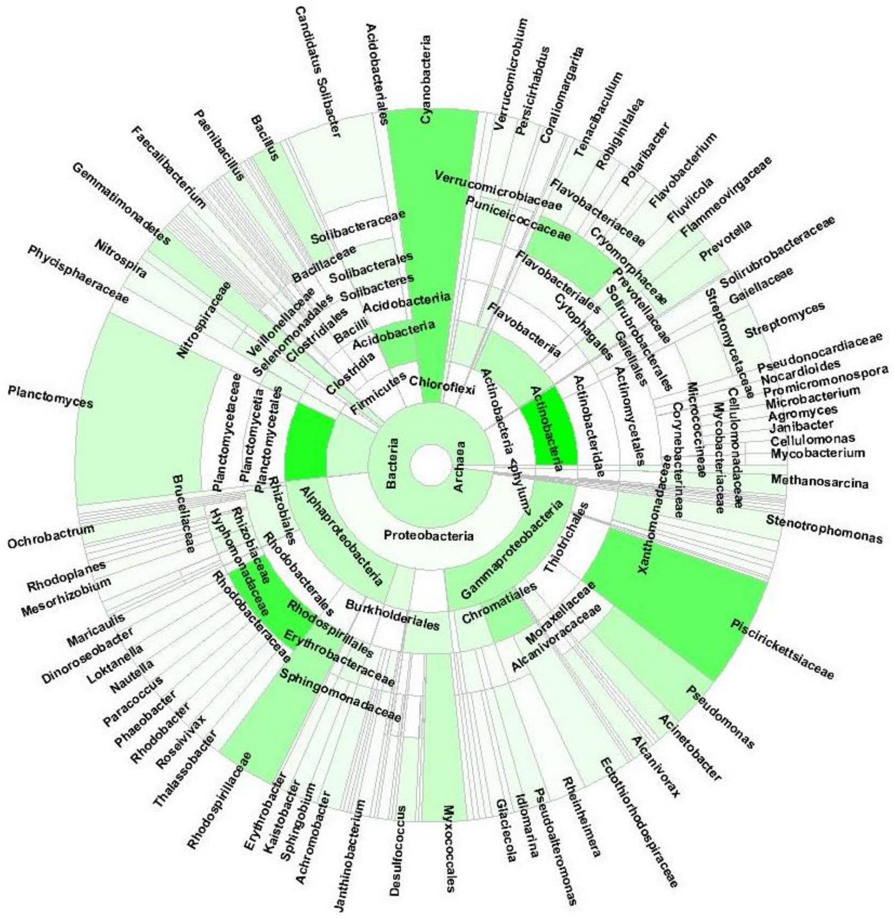

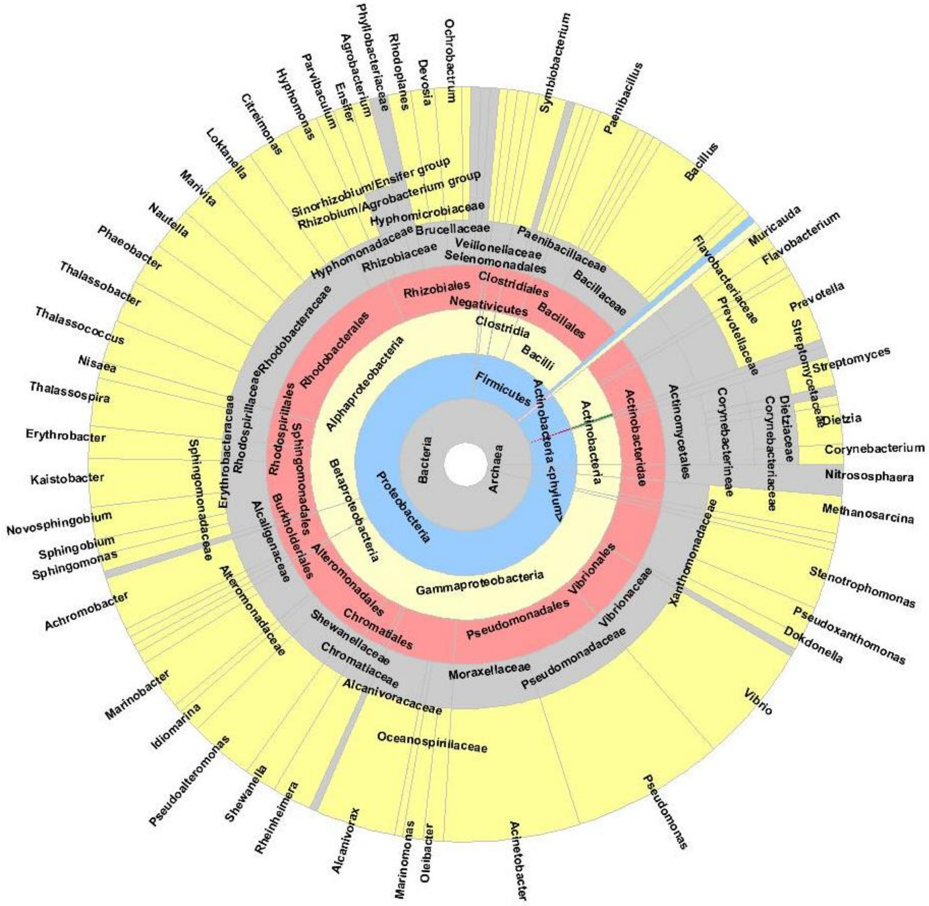

Non-oil contaminated sediment (H6)

Oil contaminated sediment (H2)

Supplementary Figure 5: Bacterial abundance at Genus level in enrichment (H1)

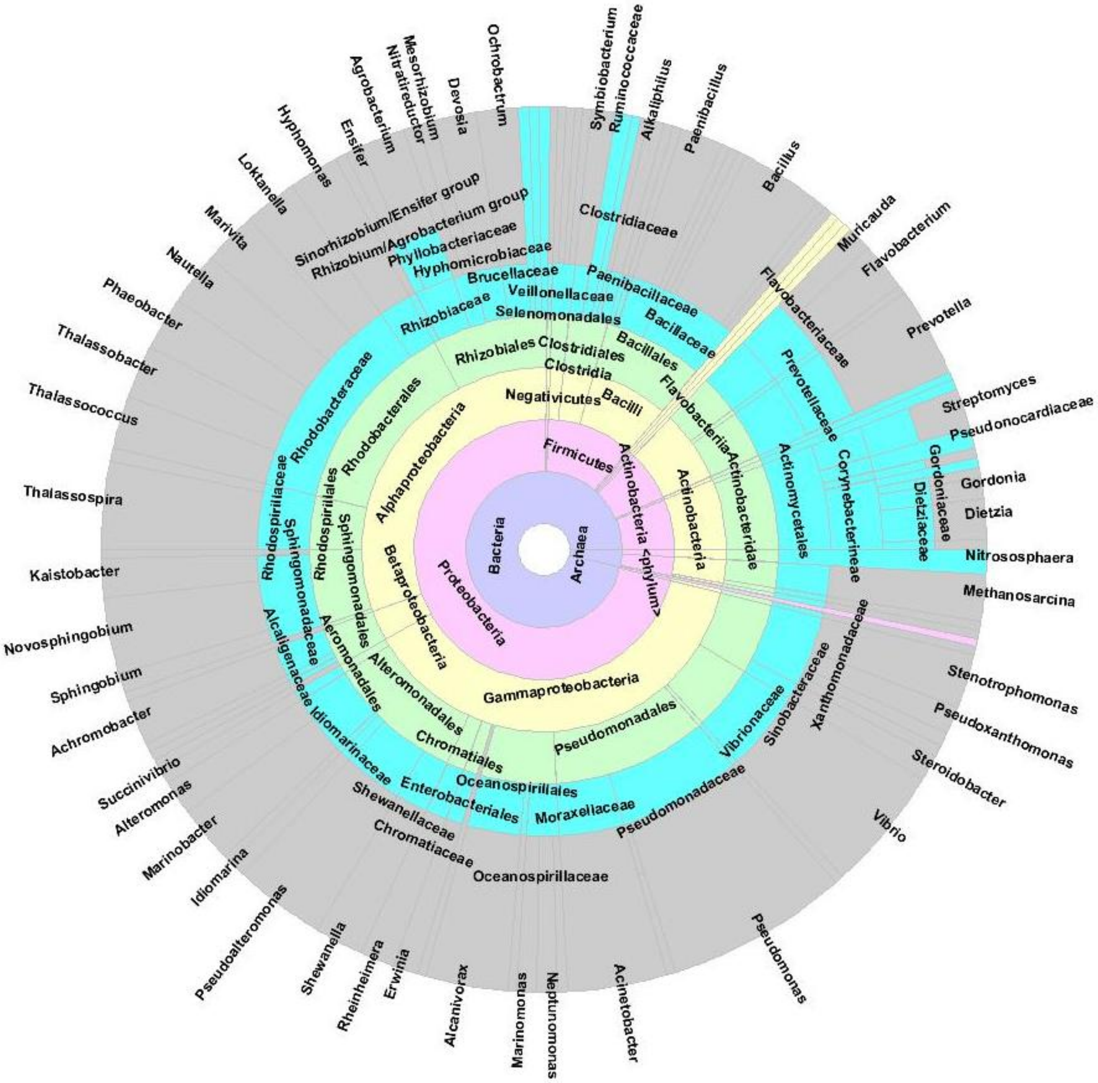

Laboratory Enrichment (H1)

Supplementary Figure 6: Relative abundance of top 25 bacterial orders among various samples based on absolute OTU counts

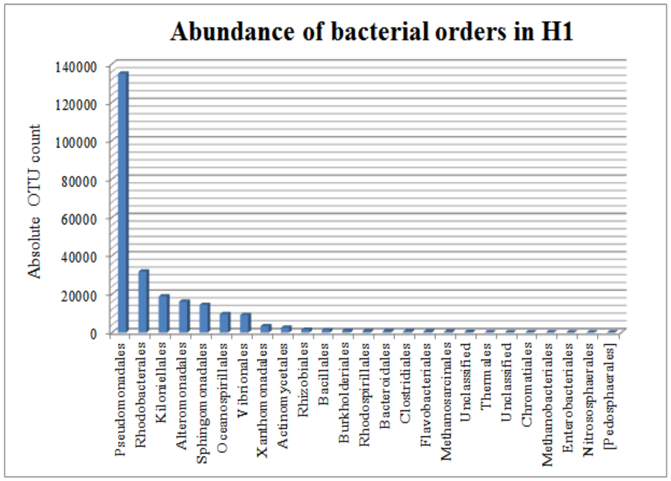

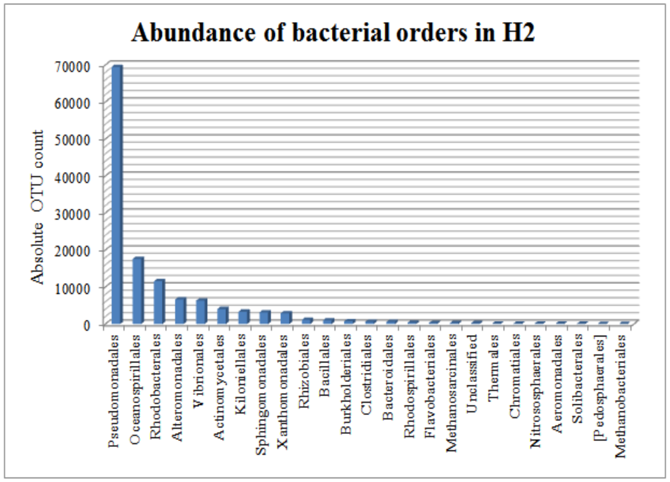

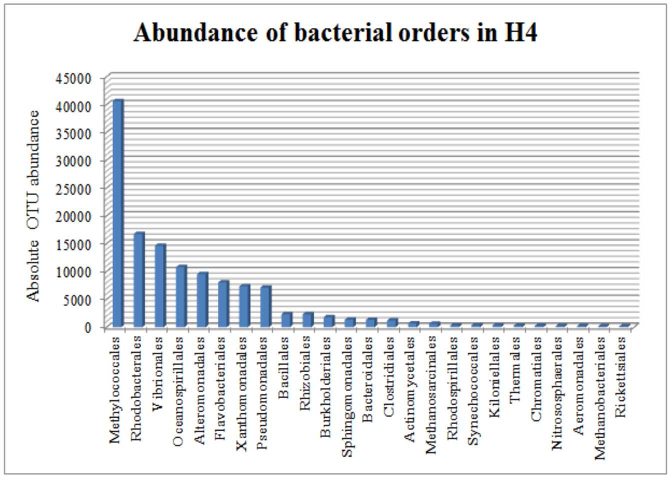

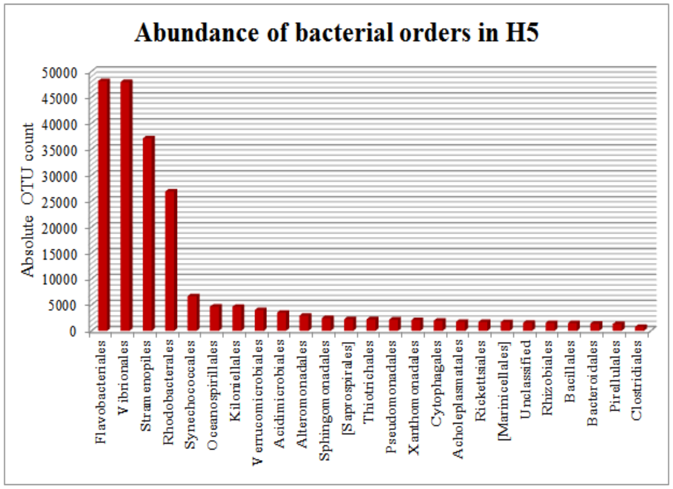

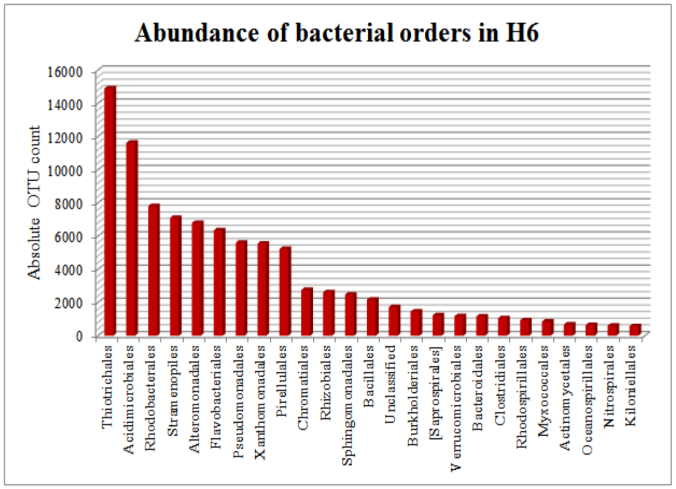

Supplementary Figure 7: Phylogenetic tree of Fungi with relative abundance of various taxonomic groups distributed among oil polluted and non-oil polluted samples represented in different colours (Violet: H6, Pink: H5 and Blue: H4)

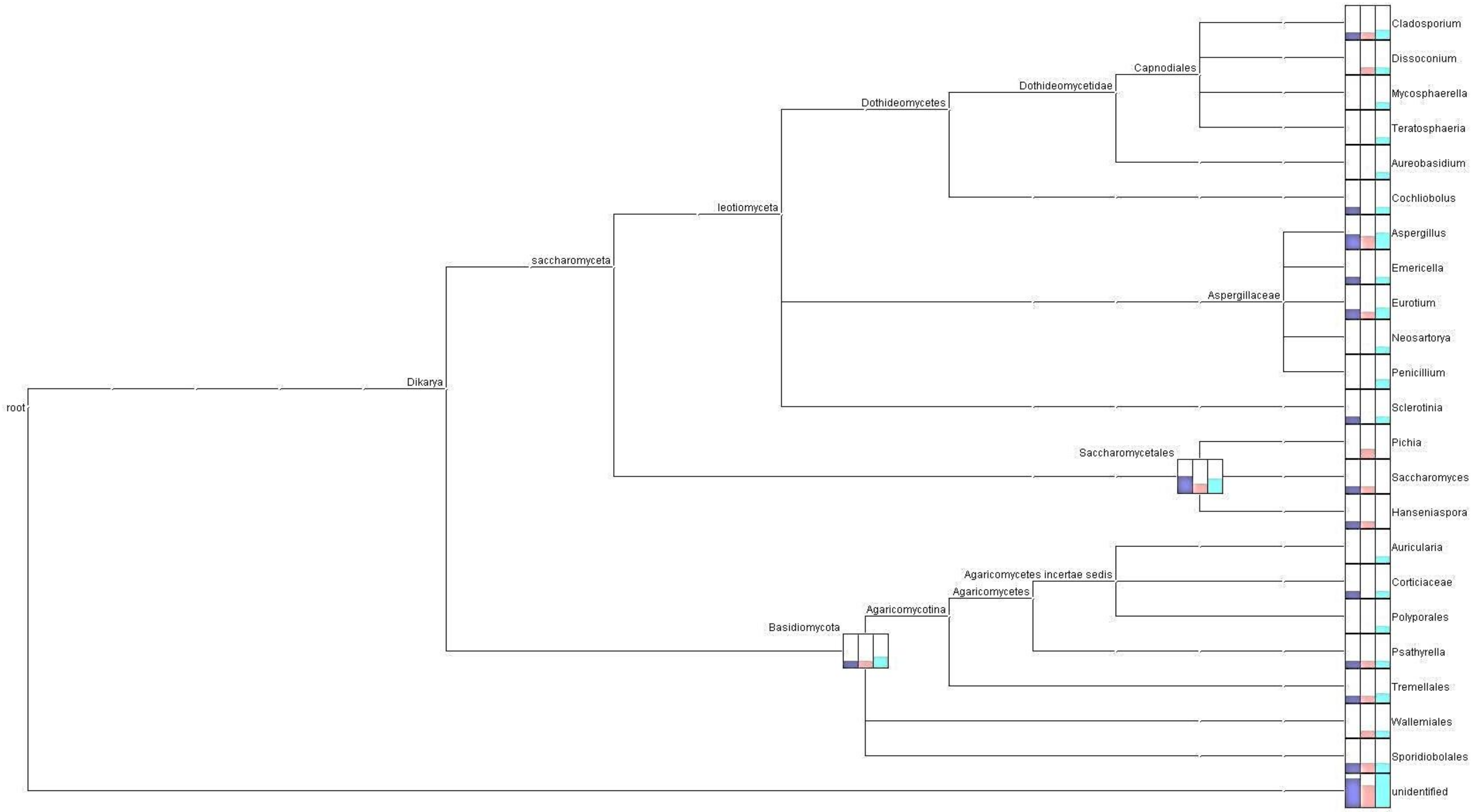

Supplementary Figure 8: Heat map of fungal distribution in among oil contaminated and non-oil contaminated samples.

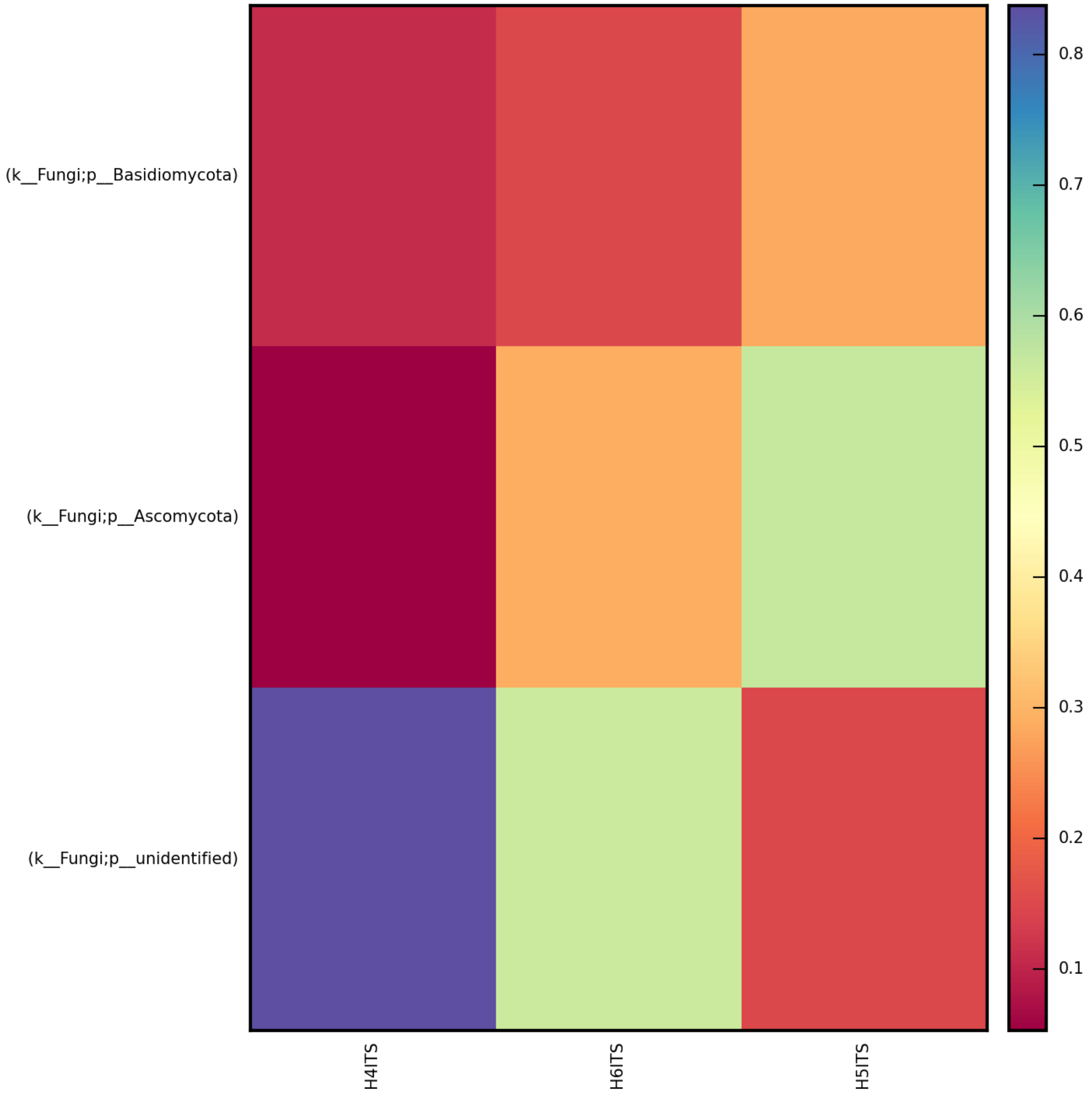

H4ITS- Oil contaminated water
H5 ITS – Non- oil contaminated water
H6 ITS – Non- oil contaminated sediment

Supplementary figure 9: Principal Component Analysis (PCA) for bacterial distribution according to their OTU $_{97}$ composition.

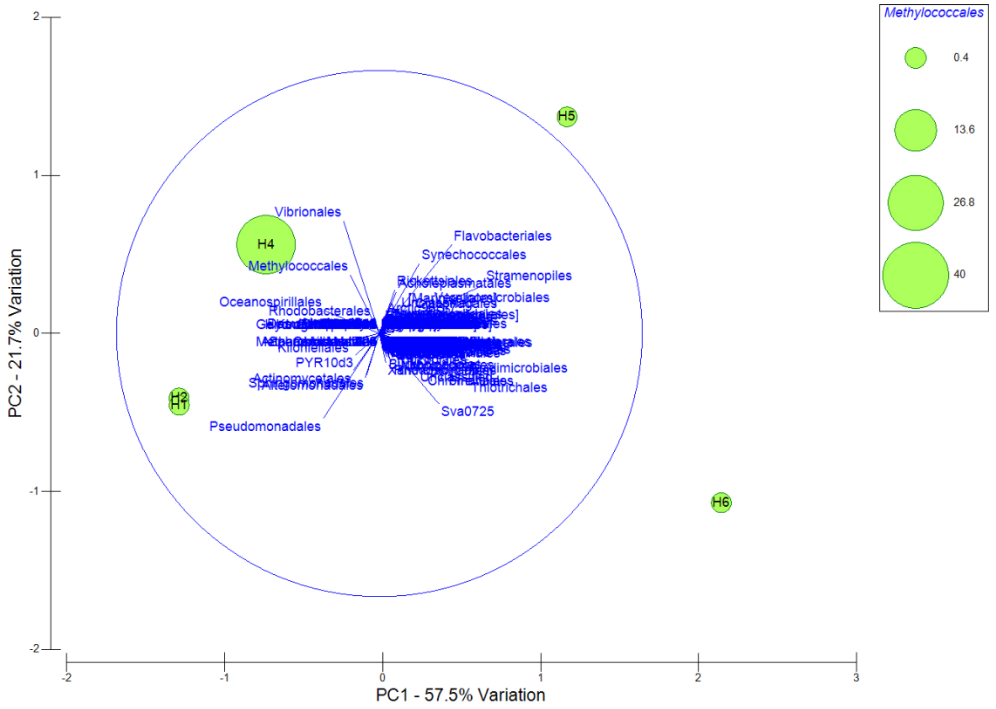

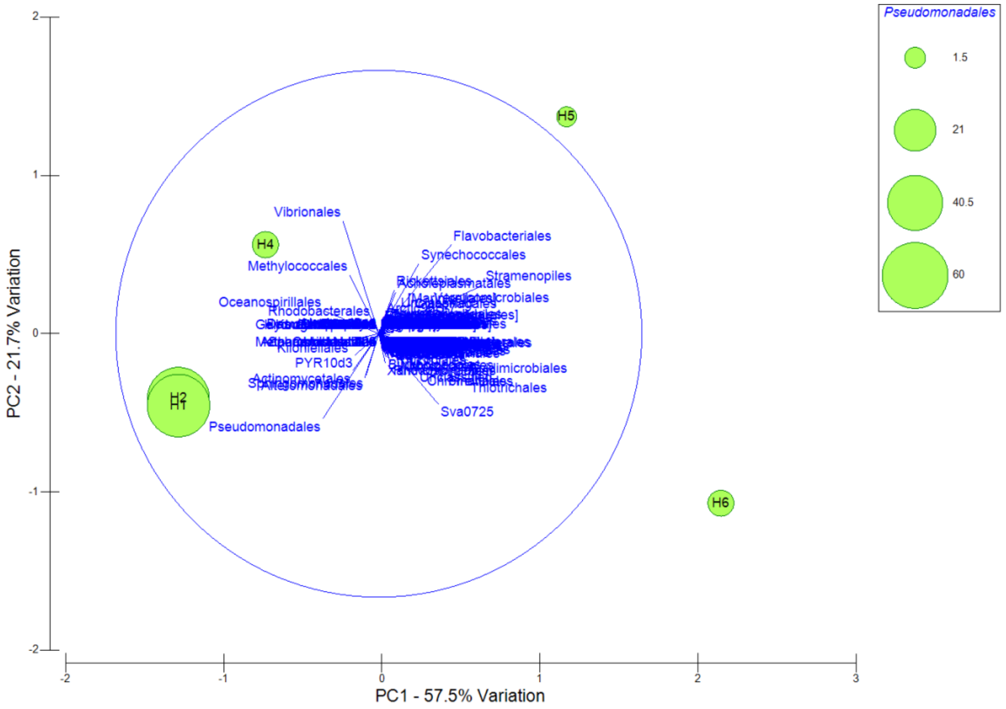

Supplementary Figure 10. Phylogenetic tree showing the affiliation of bacterial isolates with previously reported hydrocarbon degrading strains

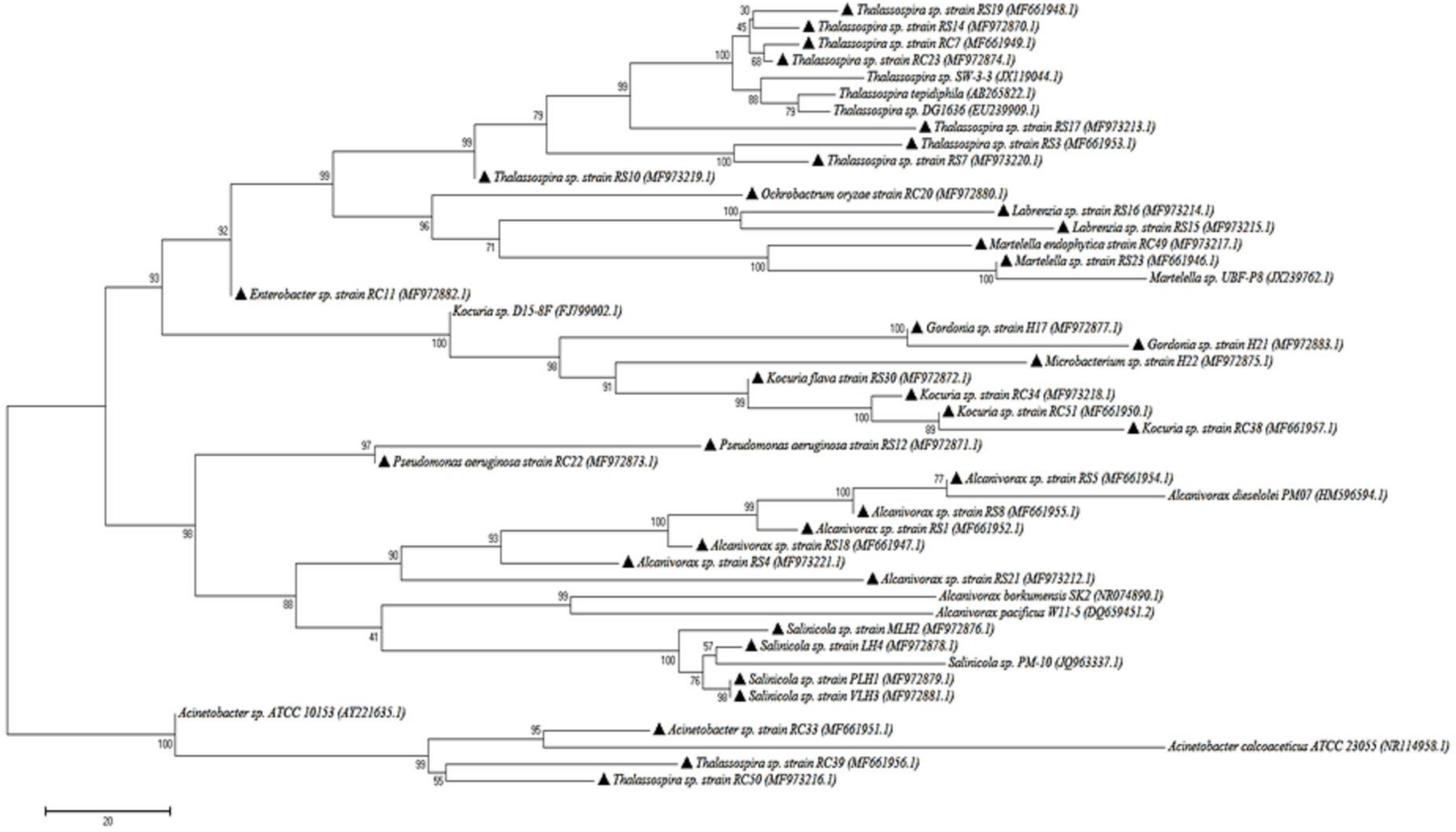

Supplementary Figure 11: Functional krona showing distribution of genes from PICRUSt analysis of microbial communities in Non oil polluted seawater (H5)

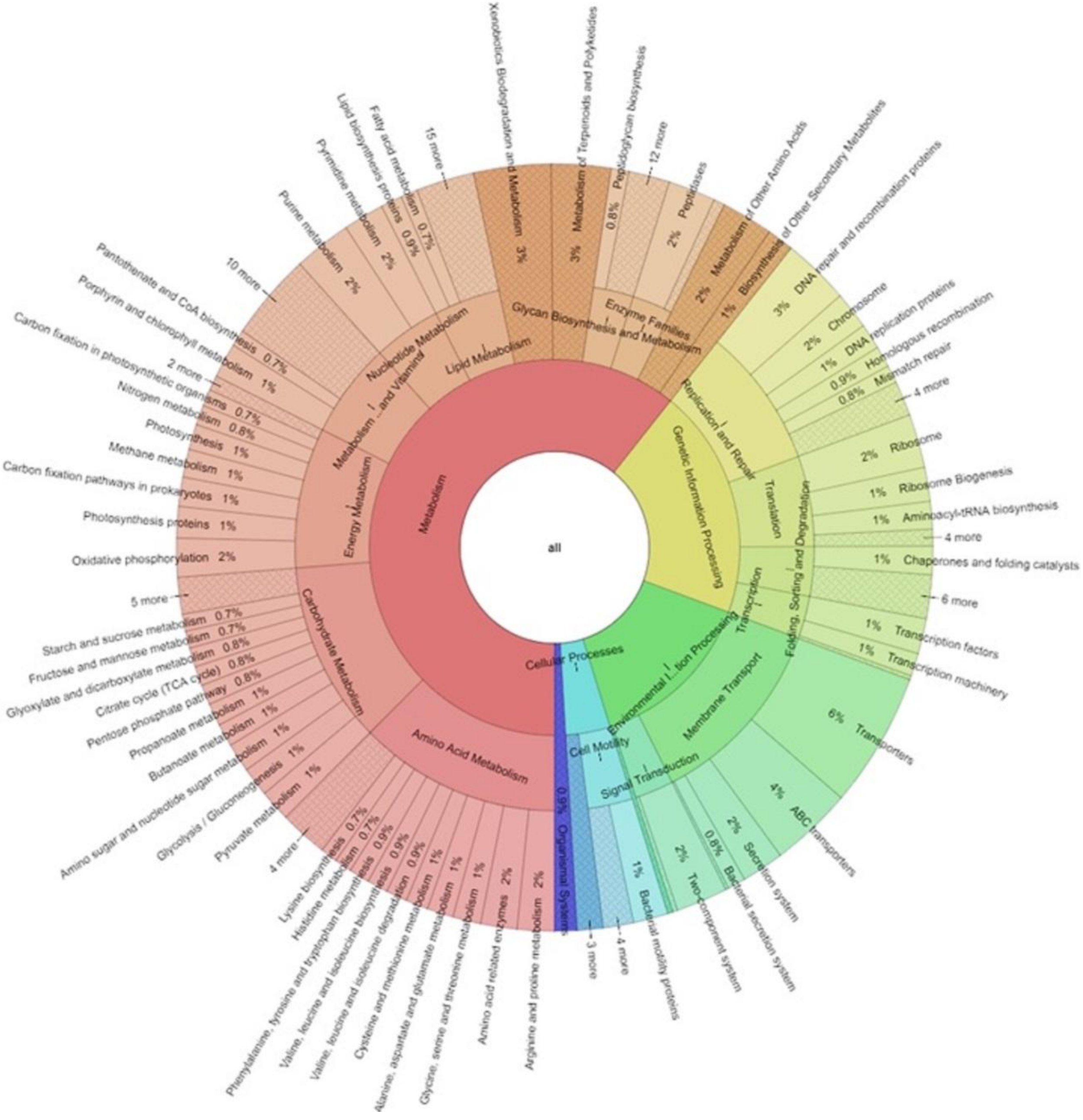

Supplementary figure 12: Functional krona showing distribution of genes from PICRUSt analysis of microbial communities in oil polluted seawater (H4)

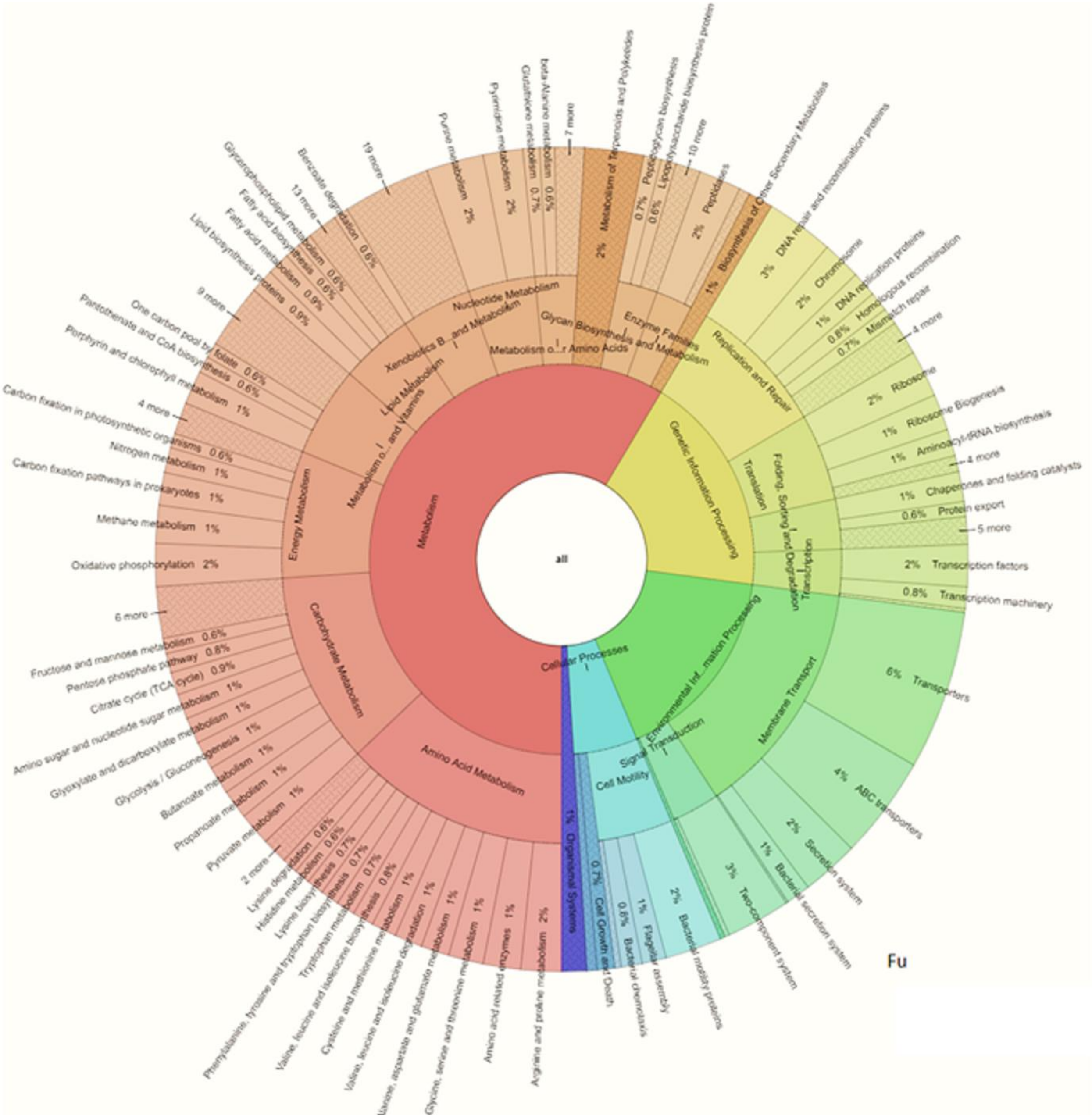

Supplementary Figure 13: Functional krona showing distribution of genes from PICRUSt analysis of microbial communities in Non-oil polluted sediment (H6)

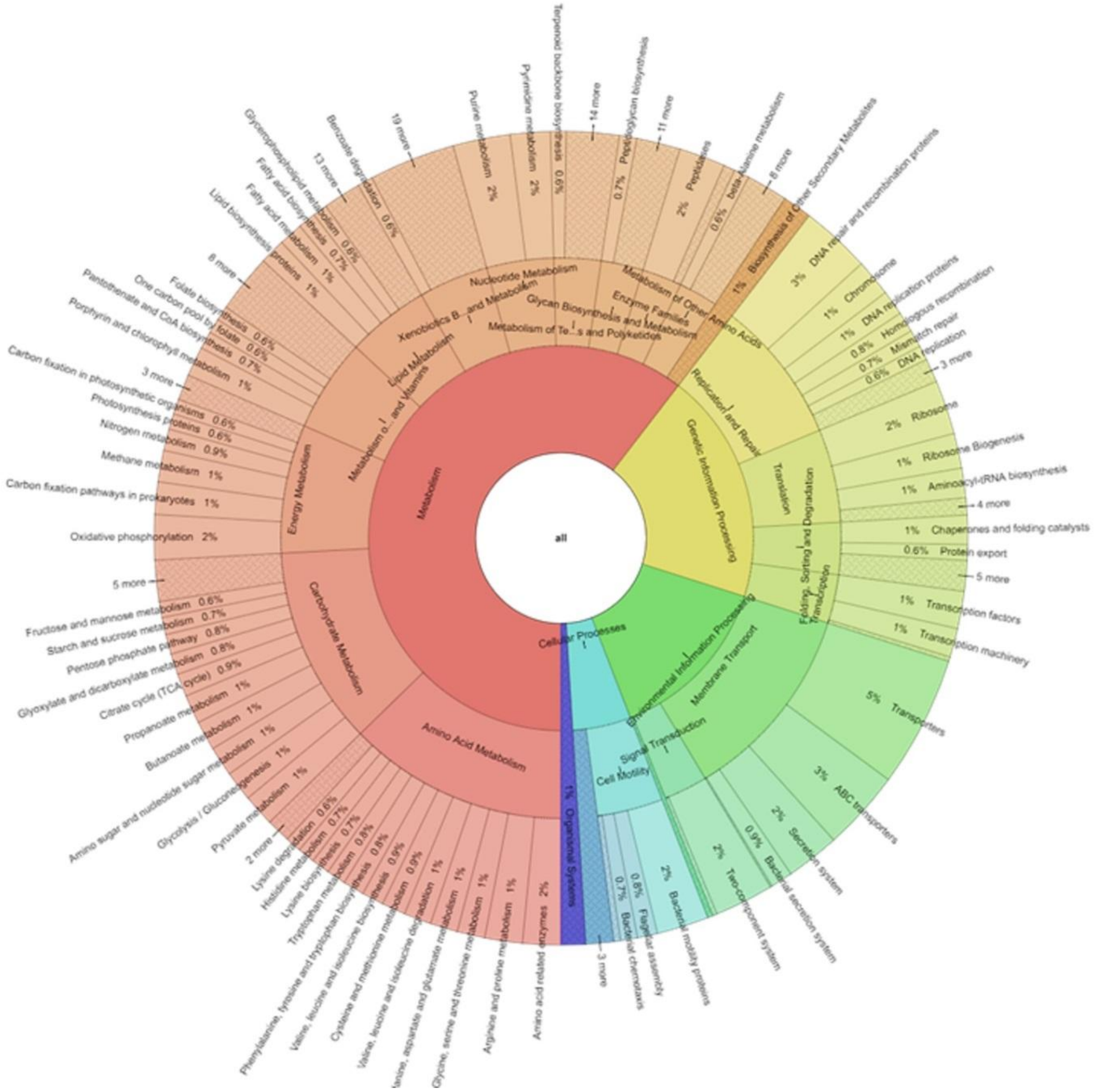

Supplementary Figure 14: Functional krona showing distribution of genes from PICRUSt analysis of microbial communities in oil polluted sediment (H2)

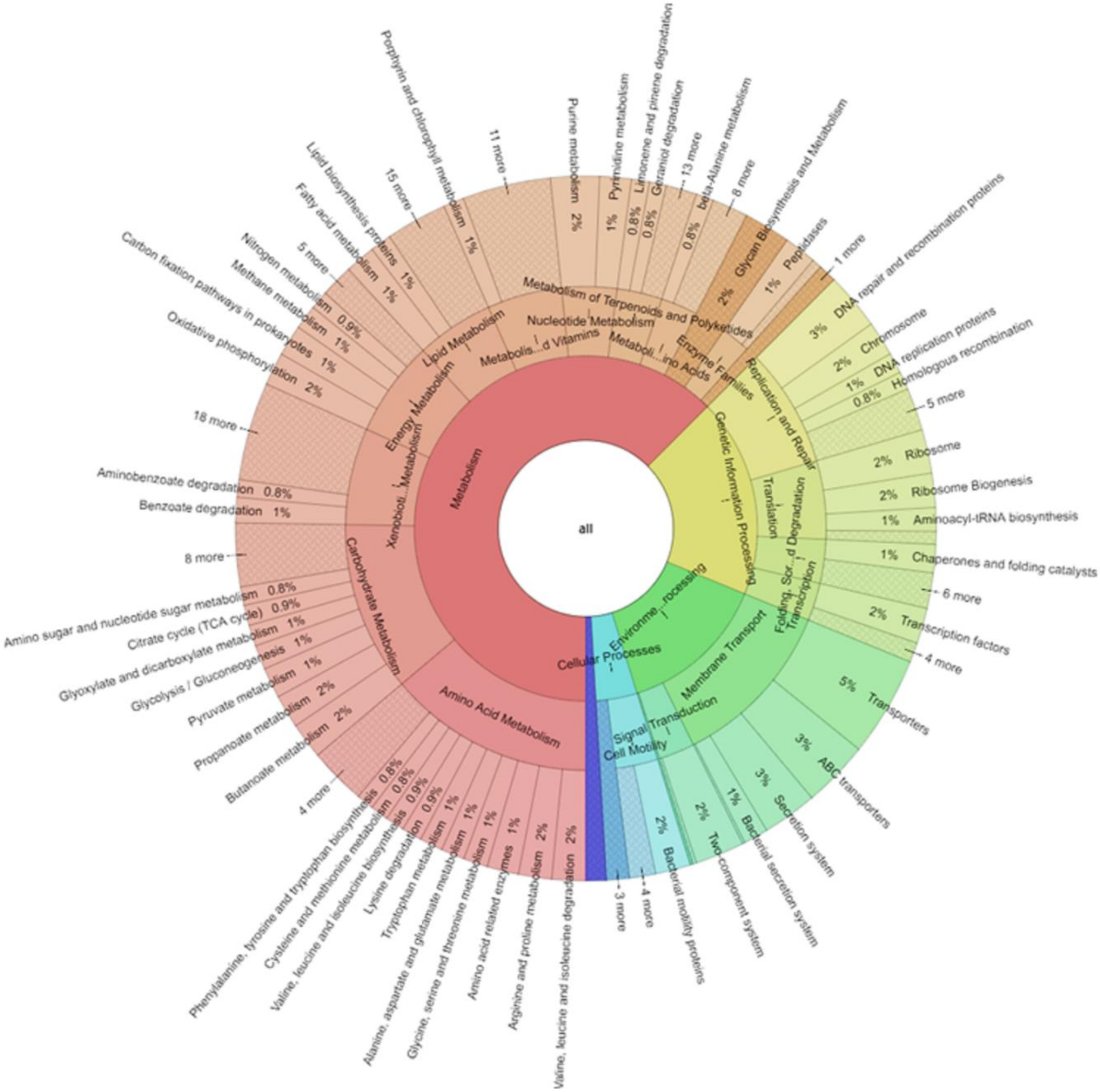

Supplementary Figure 15: Functional krona showing distribution of genes from PICRUSt analysis of microbial communities in laboratory enrichment (H1)

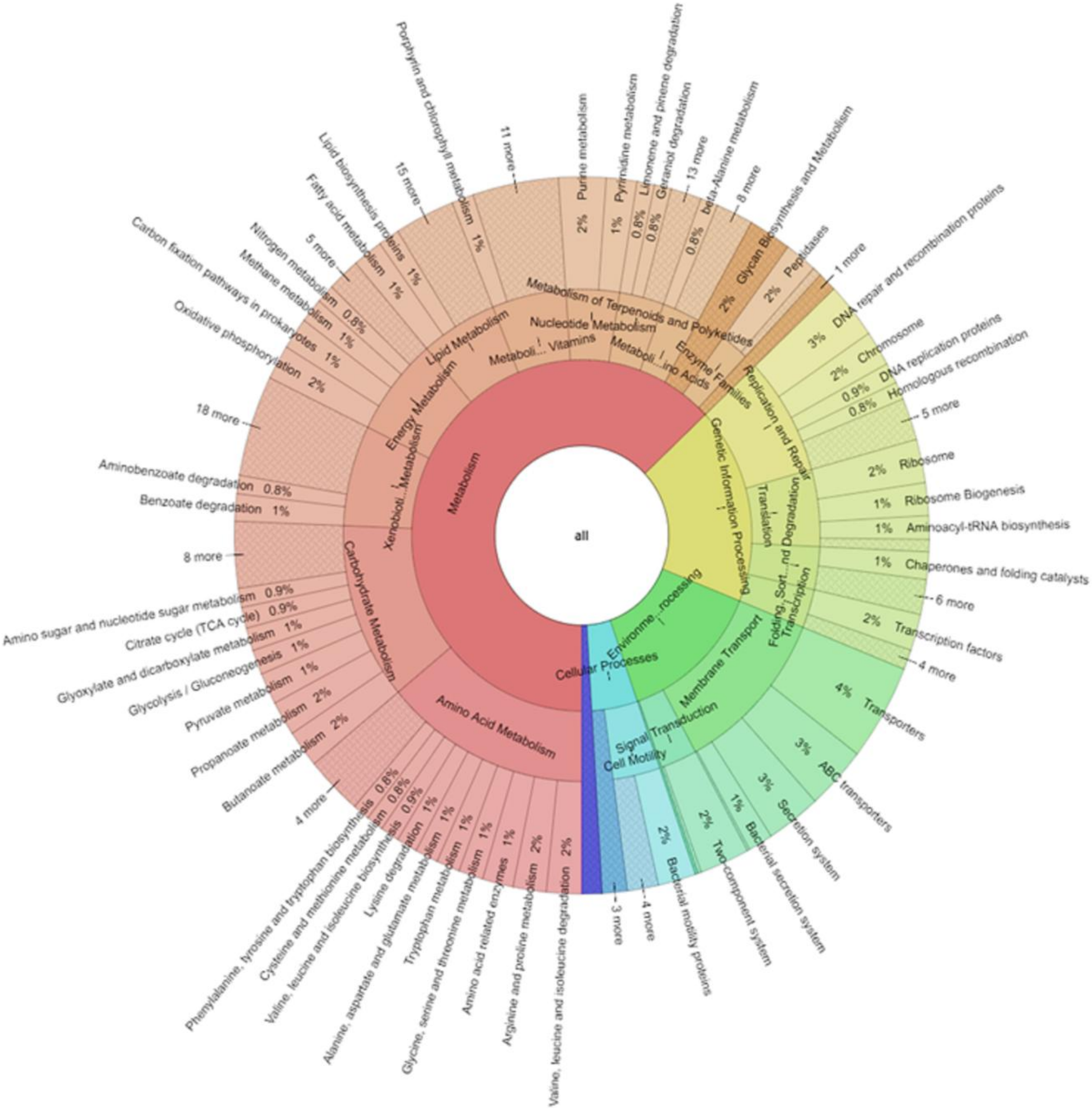

Supplementary Figure 16: KEGG pathway showing key catabolic network of benzoate biodegradation. Forty-five different enzymes involved in benzoate degradation were observed in different samples and are represented in blue coloured nodes. Bar plots inside the nodes correspond to the relative abundance of enzymes between the samples (H1, H2, H4, H5 and H6). The KEGG pathway[5] map 00362 is adapted here from http://www.kegg.jp/kegg/kegg1.html. and enriched with data using VANTED (V2.2.1)

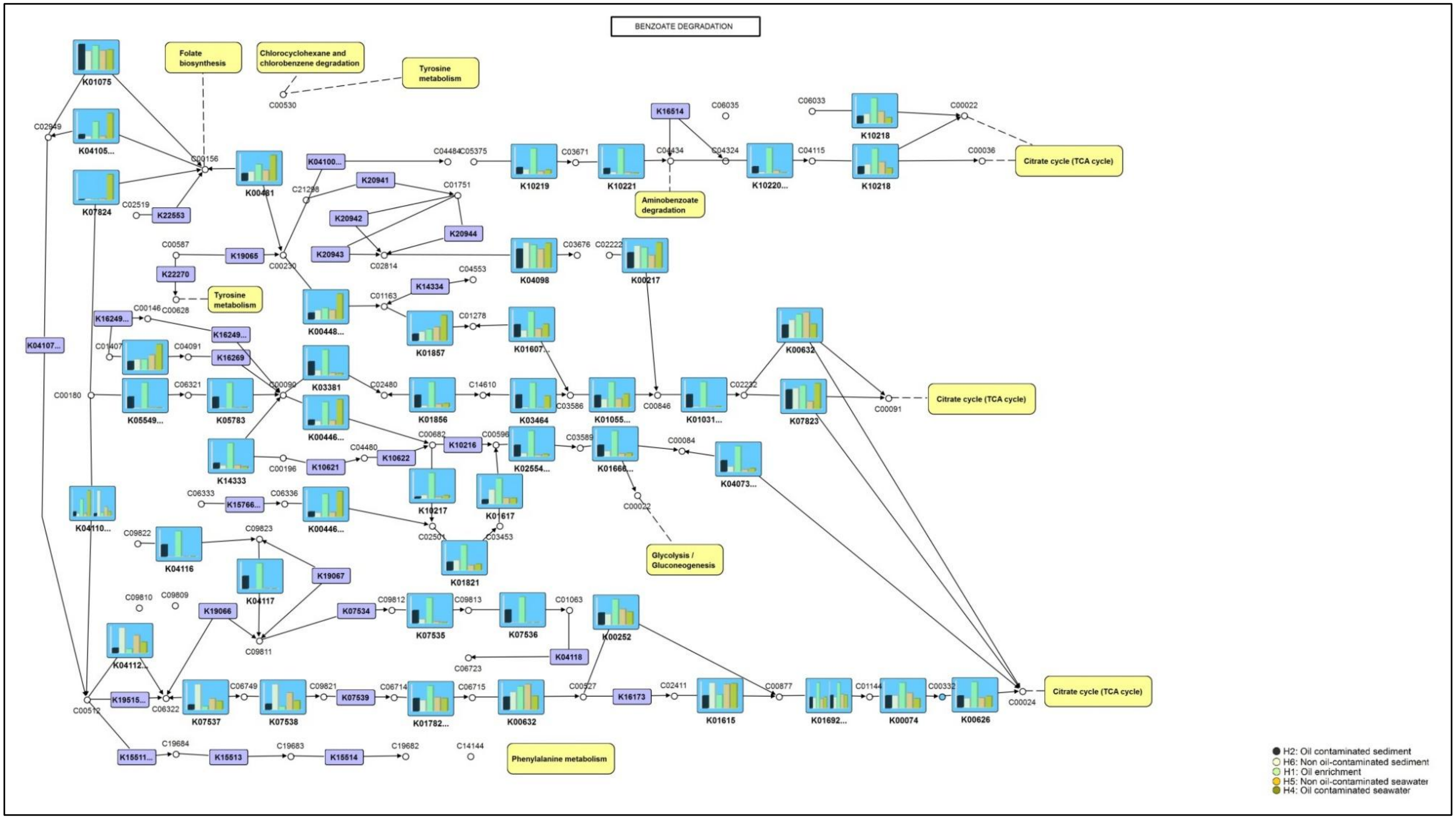

Supplementary Figure 17: KEGG pathway showing key catabolic network of toluene biodegradation. Twelve different enzymes involved in toluene degradation were observed in different samples and are represented in pink coloured nodes. Bar plots inside the nodes correspond to the relative abundance of enzymes between the samples (H1, H2, H4, H5 and H6). The KEGG pathway[5] map 00623 is adapted here from http://www.kegg.jp/kegg/kegg1.html. and enriched with data using VANTED (V2.2.1)

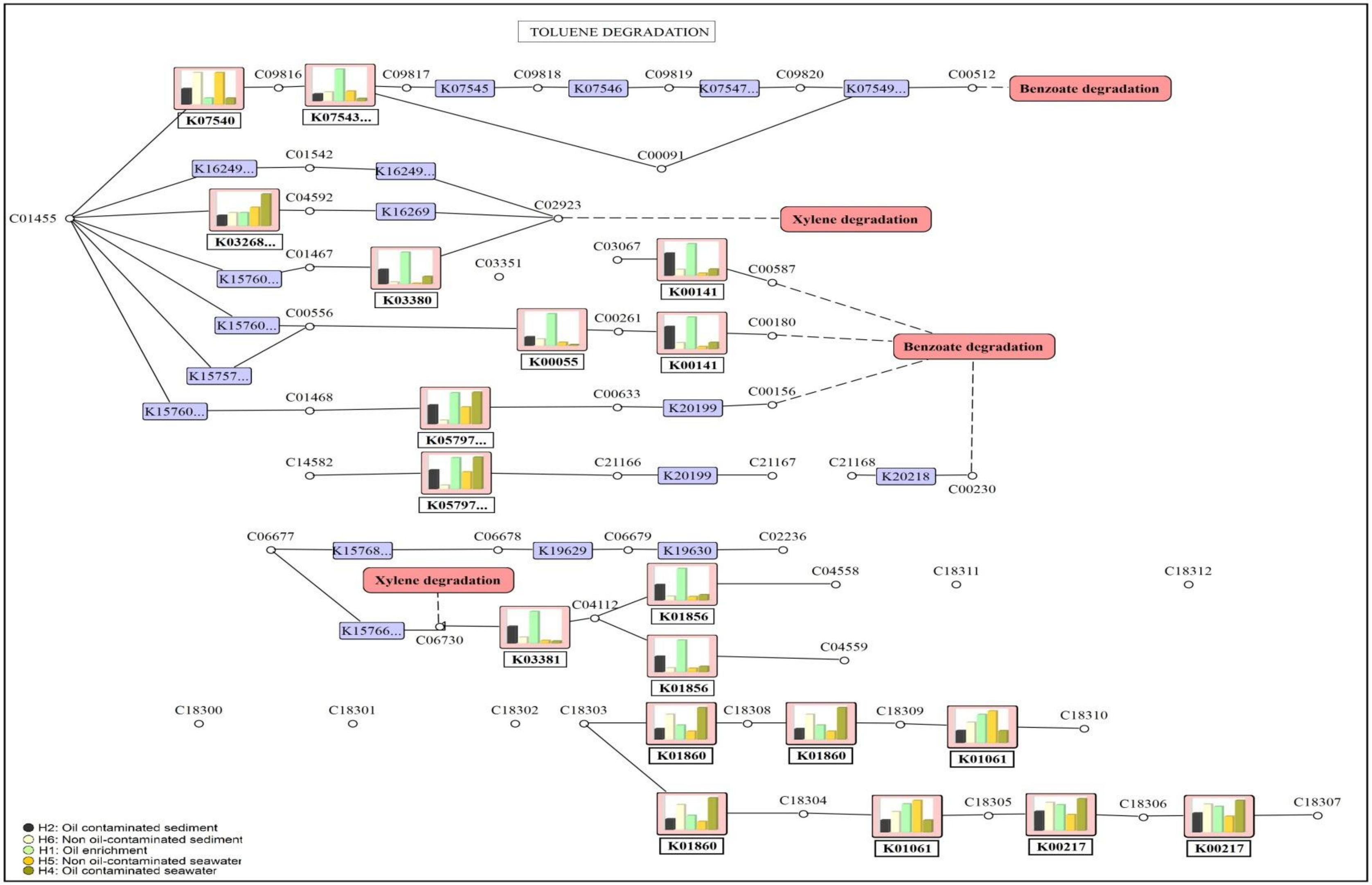

Supplementary Figure 18: KEGG pathway showing key catabolic network of xylene biodegradation. Fifteen different enzymes involved in xylene degradation were observed in different samples and are represented in grey coloured nodes. Bar plots inside the nodes correspond to the relative abundance of enzymes between the samples (H1, H2, H4, H5 and H6). The KEGG pathway[5] map 00622 is adapted here from http://www.kegg.jp/kegg/kegg1.html. and enriched with data using VANTED (V2.2.1)

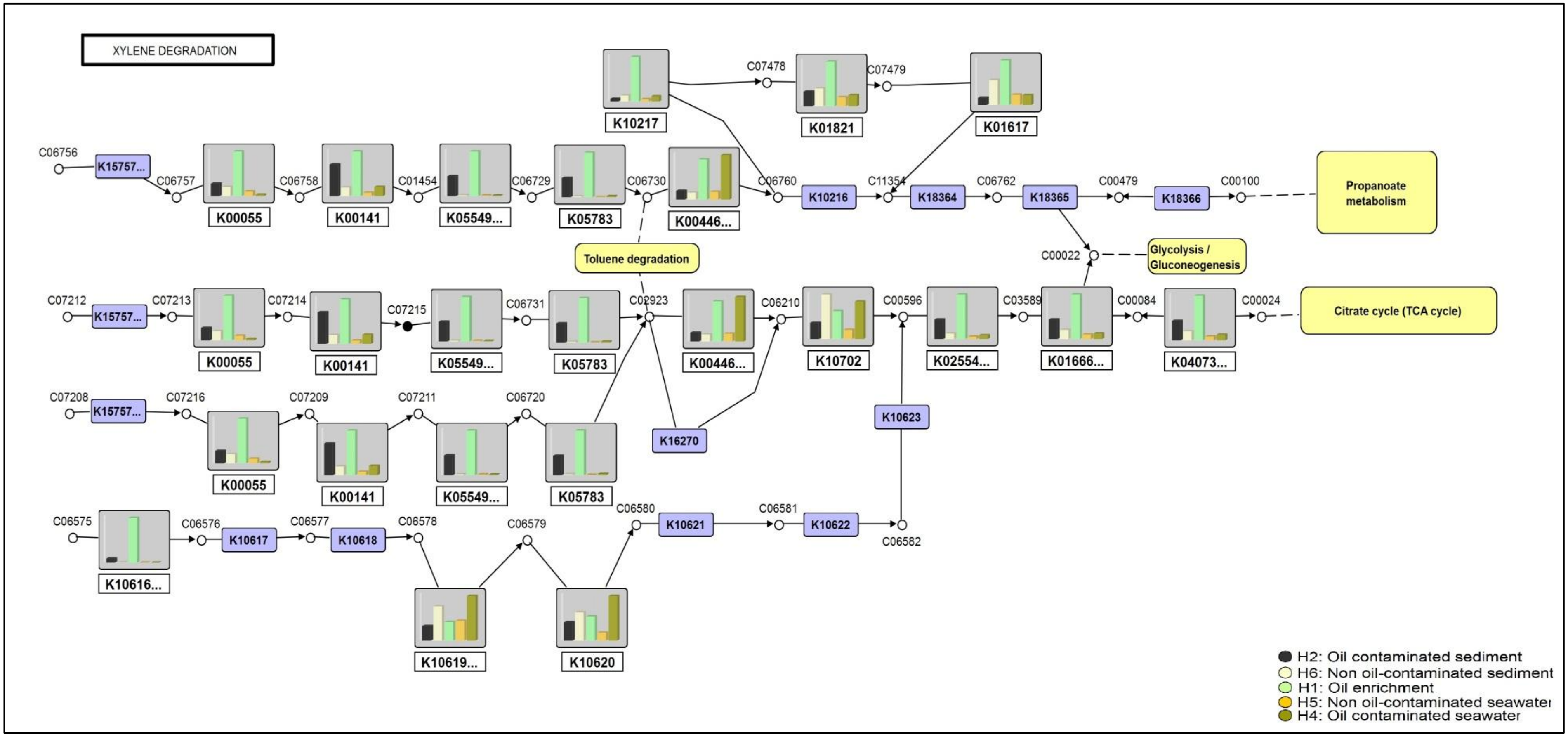

Supplementary Figure 19: Potential enhancement and reduction in genes among different samples from PICRUSt analysis of microbial communities

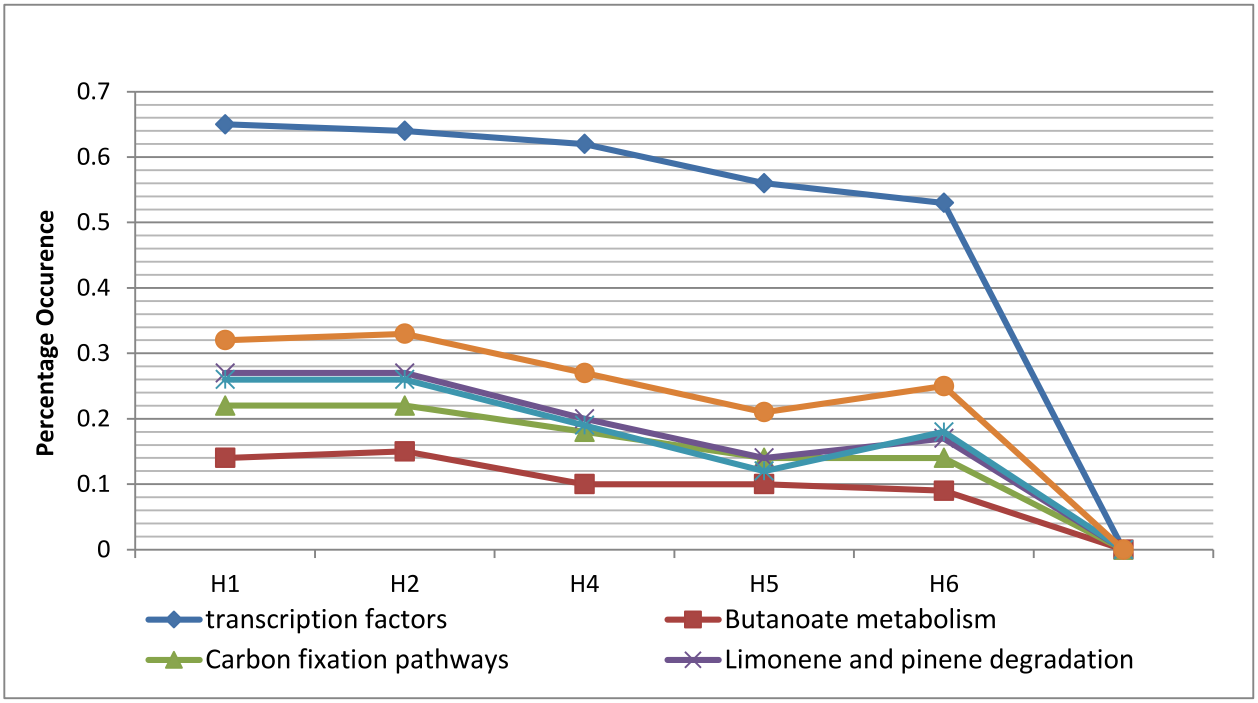

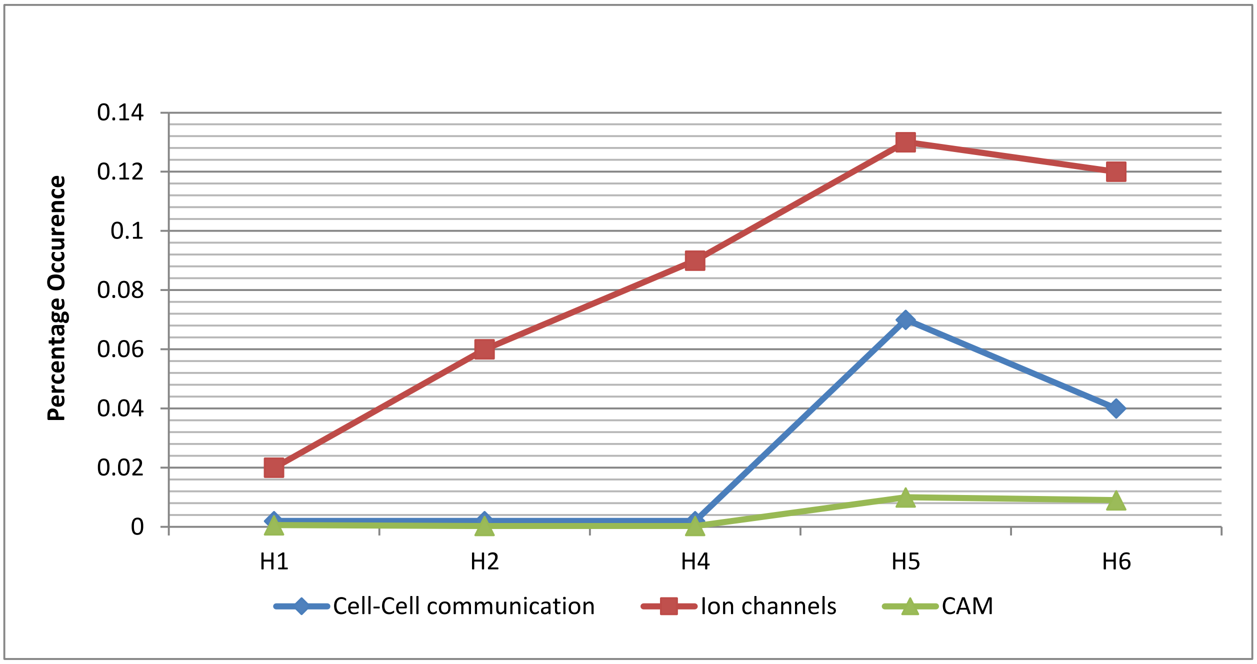

Supplementary Figure 20: Polycyclic Aromatic Hydrocarbon (PAH) content analysis of various enrichments using Gas chromatography-Mass spectroscopy

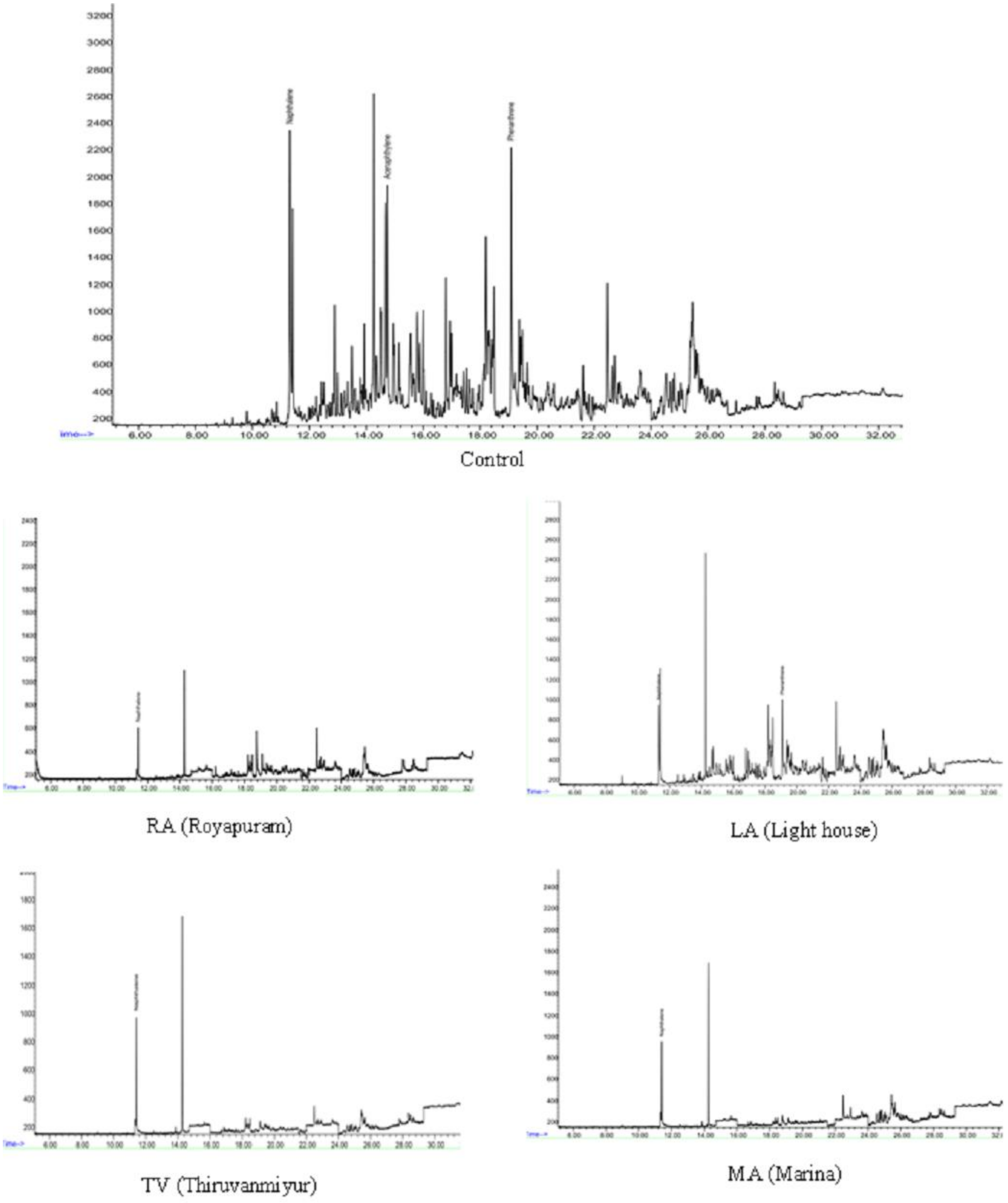

Supplementary Figure 21: Total Petroleum Hydrocarbon (TPH) content analysis of various enrichments using Gas chromatography-Mass spectroscopy

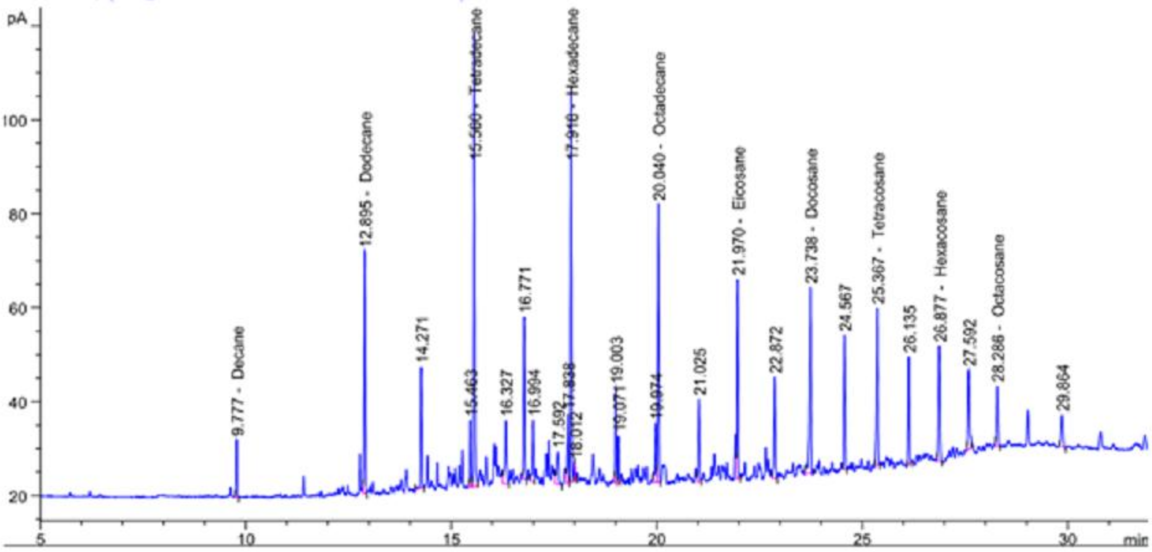

Control

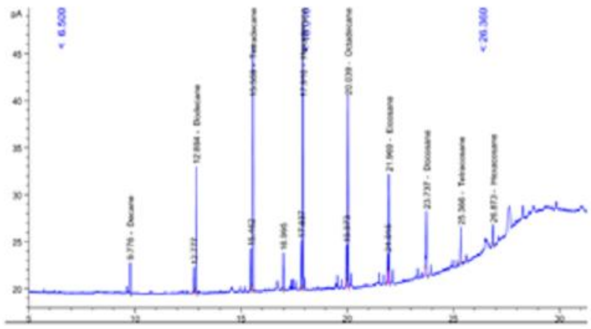

Light House (LA)

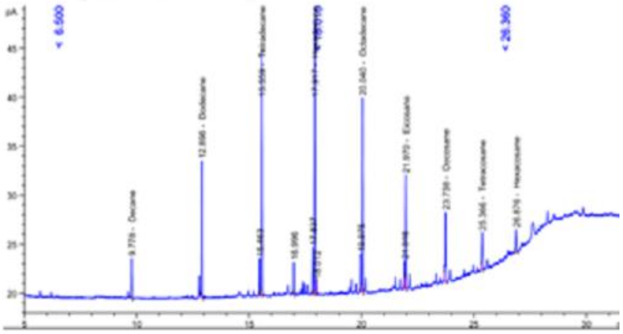

Royapuram (RA)

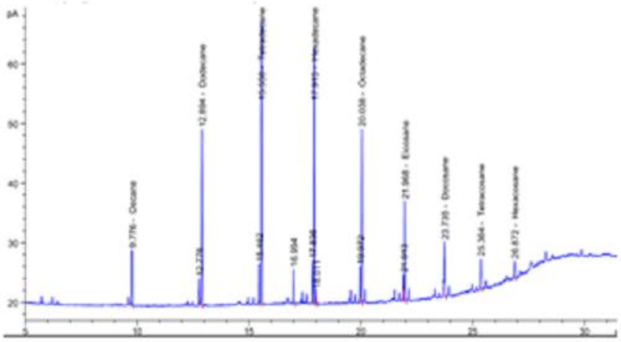

Marina (MA)

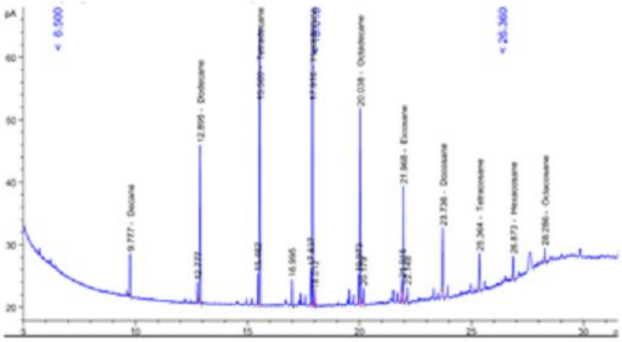

Thiruvanmiyur (TV)

Chromatogram of CHNS (Elemental Analyzer)

BBOT NCHS (Based on chromatogram retention time) Standard

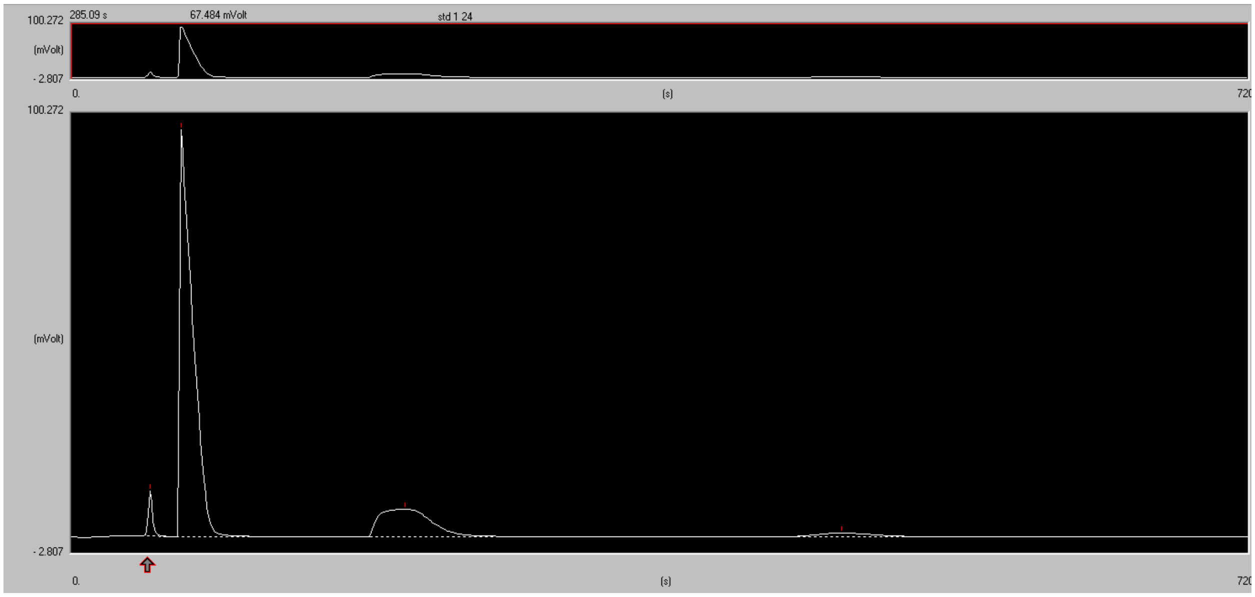

Chromatogram of CHNS in sediment sample- Before spill

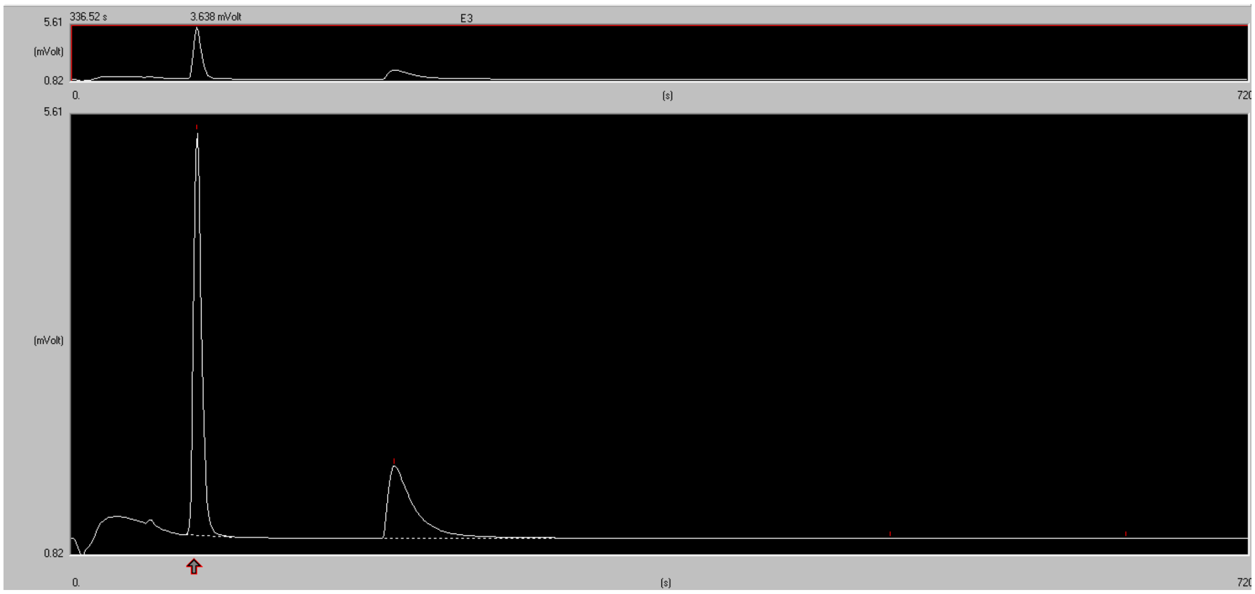

Chromatogram of Carbon in sediment sample-After spill

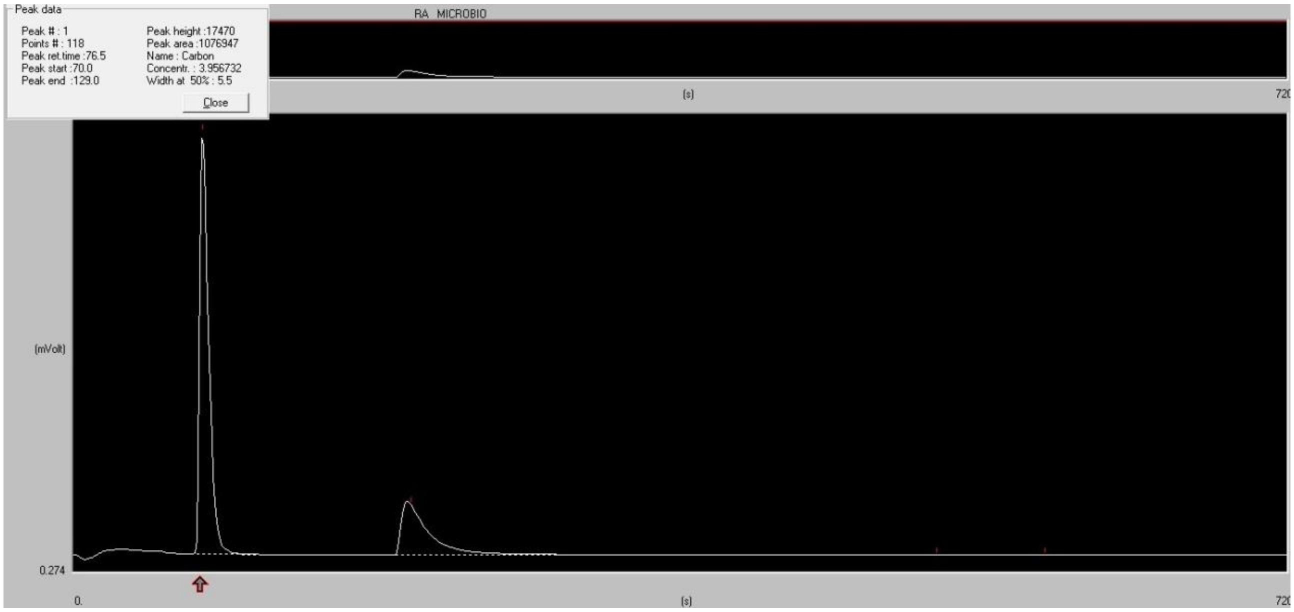

Chromatogram of Nitrogen in sediment sample-After spill

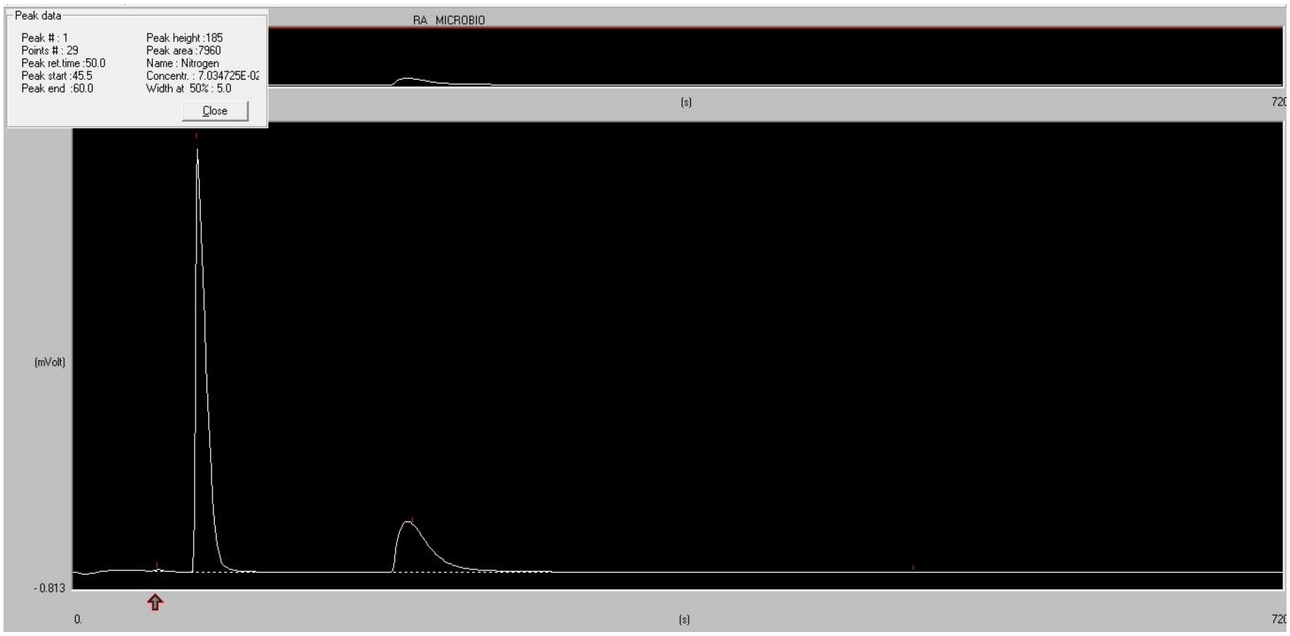

Chromatogram of Hydrogen in sediment sample-After spill

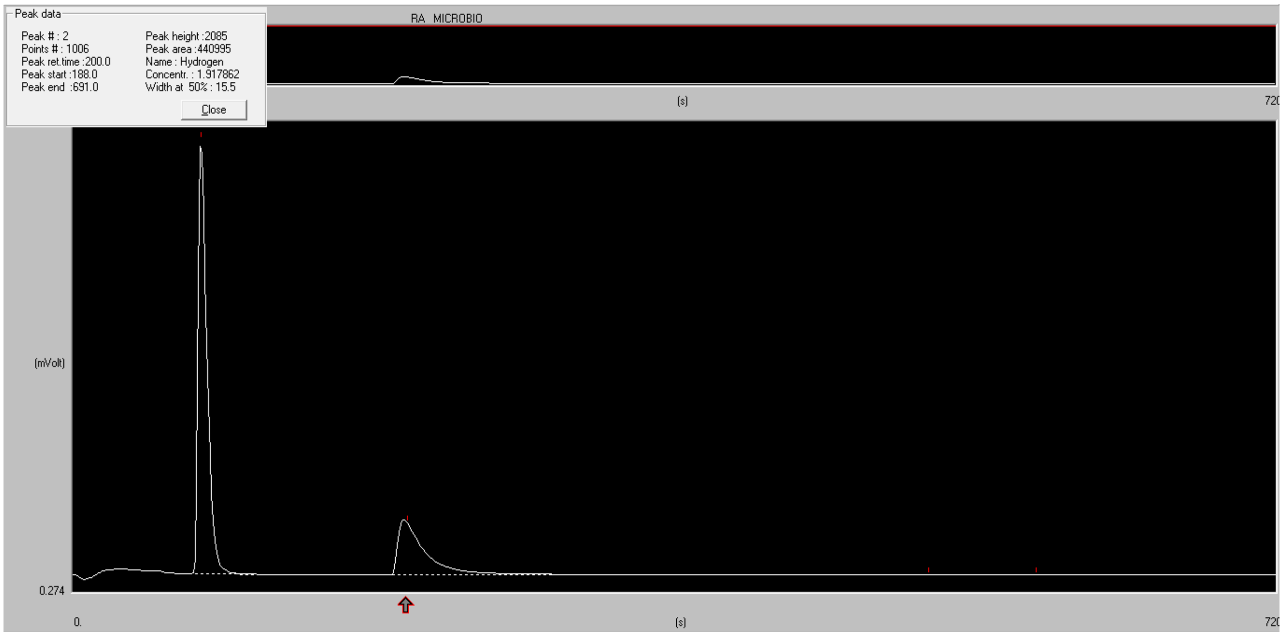

Chromatogram of Sulphur in sediment sample-After spill

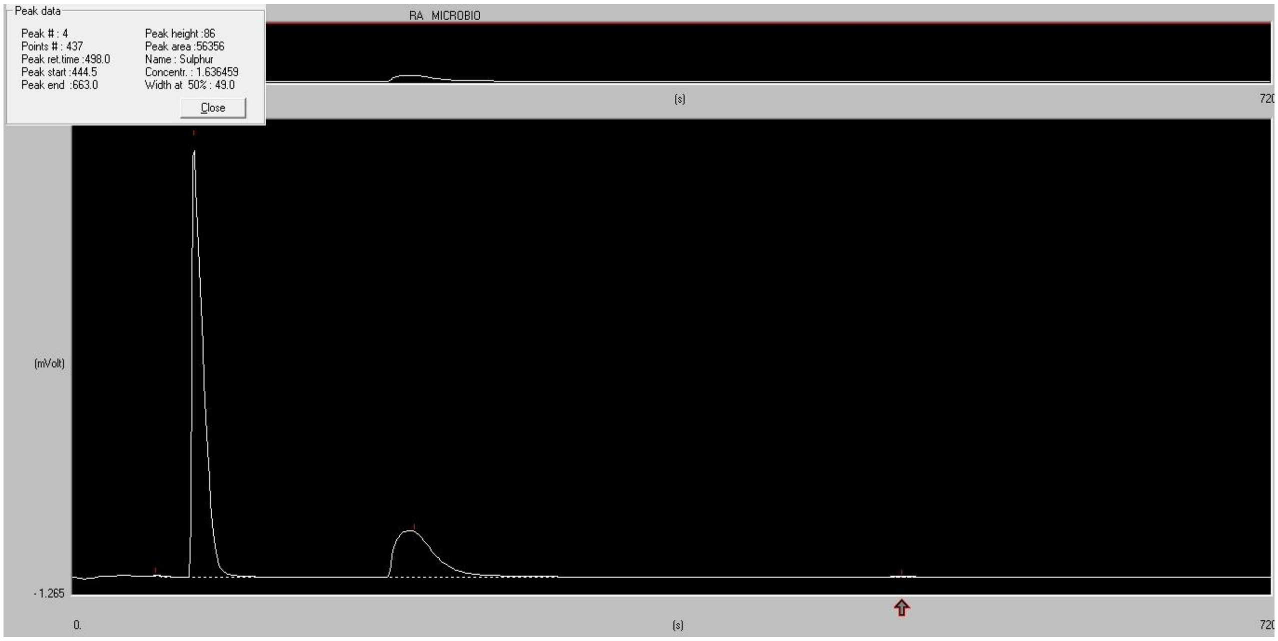

Supplementary Reference

1. Weisburg, W.G., Barns, S.M., Pelletier, D.A., Lane, D.J. 16S ribosomal DNA amplification for phylogenetic study. *J. Bacteriol.* **173**, 697-703 (1991).
2. Kuhn, E., Bellicanta, G.S., Pellizari, V.H. New *alk* genes detected in Antarctic marine sediments. *Environ Microbiol* **11**, 669–673 (2009).
3. Klindworth, A. *et al*. Evaluation of general 16S ribosomal RNA gene PCR primers for classical and next-generation sequencing-based diversity studies. *Nucleic Acids Res*. **41**, e1. (2013).
4. White, T. J., T. D. Bruns, S. B. Lee, and J. W. Taylor. Amplification and direct sequencing of fungal ribosomal RNA Genes for phylogenetics. In PCR - A guide to methods and applications (eds. MA Innis, DH Gelfand, JJ Sninsky and TJ White). 315-322 (Academic Press, San Diego, 1990).
5. Kanehisa, F.M., Tanabe, M., Sato, Y., Morishima, K.; KEGG: new perspectives on genomes, pathways, diseases and drugs. *Nucleic Acids Res*. **45**, D353-D361 (2017).
